# Promastigote EPS secretion and haptomonad biofilm formation as evolutionary adaptations of trypanosomatid parasites for colonizing honeybee hosts

**DOI:** 10.1038/s41522-024-00492-x

**Published:** 2024-03-21

**Authors:** Jéssica Carreira de Paula, Pedro García Olmedo, Tamara Gómez-Moracho, María Buendía-Abad, Mariano Higes, Raquel Martín-Hernández, Antonio Osuna, Luis Miguel de Pablos

**Affiliations:** 1 Department of Parasitology, Biochemical and Molecular Parasitology Group CTS-183, University of Granada, Granada, Spain; 2 Institute of Biotechnology, University of Granada, Granada, Spain; 3 Laboratorio de Patología Apícola, Centro de Investigación Apícola y Agroambiental (CIAPA), IRIAF – Instituto Regional de Investigación y Desarrollo Agroalimentario y Forestal, Consejería de Agricultura de la Junta de Comunidades de Castilla-La Mancha, Marchamalo, Spain; 4 Instituto de Recursos Humanos para la Ciencia y la Tecnología, Fundación Parque Científico y Tecnológico de Castilla-La Mancha, 02006 Albacete, Spain

**Keywords:** Pathogens, Biofilms

## Abstract

Bees are major pollinators involved in the maintenance of all terrestrial ecosystems. Biotic and abiotic factors placing these insects at risk is a research priority for ecological and agricultural sustainability. Parasites are one of the key players of this global decline and the study of their mechanisms of action is essential to control honeybee colony losses. Trypanosomatid parasites and particularly the *Lotmaria passim* are widely spread in honeybees, however their lifestyle is poorly understood. In this work, we show how these parasites are able to differentiate into a new parasitic lifestyle: the trypanosomatid biofilms. Using different microscopic techniques, we demonstrated that the secretion of Extracellular Polymeric Substances by free-swimming unicellular promastigote forms is a prerequisite for the generation and adherence of multicellular biofilms to solid surfaces in vitro and in vivo. Moreover, compared to human-infective trypanosomatid parasites our study shows how trypanosomatid parasites of honeybees increases their resistance and thus resilience to drastic changes in environmental conditions such as ultralow temperatures and hypoosmotic shock, which would explain their success thriving within or outside their hosts. These results set up the basis for the understanding of the success of this group of parasites in nature and to unveil the impact of such pathogens in honeybees, a keystones species in most terrestrial ecosystems.

## Introduction

The differentiation into biofilm multicellular communities is a survival strategy employed by a wide number of microorganisms to thrive under extremely harsh conditions and changing environments. These multicellular matrixes are embedded by a self-produced matrix of Extracellular Polymeric Substances (EPS) composed of a complex mixture of water-soluble-polysaccharides, nucleic acids (extracellular DNA and RNA), proteins, lipids and other biomolecules^1^. The EPS secretion is primarily produced by the free-swimming non-adherent unicellular flagellated stage of the microorganism and serves as a structural and functional scaffold for the generation of a sessile multicellular biofilm community with genuine properties such as adhesion to biotic and abiotic surfaces, tolerance to antimicrobials, resistance to environmental changes (e.g. pH, osmotic pressure, UV), intercellular communication, entrapment of nutrients and/or as carbon source^2–4^. This life cycle strategy is considered to be one of the most widely distributed and successful modes of life on Earth^2^. It involves three stages: i) EPS secretion, aggregation, and attachment; ii) growth and accumulation of the biofilm; iii) disaggregation and detachment from the biofilm^5^. Developmental differentiation into biofilms has been described in bacteria, fungi and or algae^2,6^, however, it has never been described in protistan parasites such as trypanosomatids.

Trypanosomatids (Euglenozoa: Kinetoplastea: Trypanosomatidae) are obligate eukaryotic flagellated parasites that have successfully colonized a wide range of vertebrate, invertebrate or even plant hosts^7–9^. Their life cycles could be either dixenous (two hosts) being typically transmitted by an insect vector and causing infections in vertebrates and plants or monoxenous with only one host employed for transmission and survival^8,10^. Within their hosts, the cell bodies of trypanosomatids are widely pleomorphic, with numerous forms classified and characterized by the relative position of both the nucleus and the kinetoplast (mitochondrial genome) as well as the shape and length of the flagella and cell body^11^. The majority of these parasites genera (19 out of 27) are monoxenous, infecting the digestive tract or associated organs such as salivary glands of a wide number of insects^12,13^. One of the trypanosomatid parasite forms found thriving at different locations of the insect gut is the haptomonad morphotype. The haptomonad forms are described as extracellular, non-motile, dividing, oval-shaped cells that are highly adhesive due to flagellar shortening and the formation of an adherent structure called the attachment plaque that will allow for the binding of the parasite to the surface of the foregut or hindgut of mosquitoes, flies or honeybee hosts^14–19^.

Bees are essential for the maintenance of all terrestrial ecosystems and are currently suffering a massive global decline due to a wide variety of abiotic and biotic factors where parasites play a major role^20^. To date, *Crithidia mellificae*, *C. bombi*, *C. acantocephali* and *Lotmaria passim* trypanosomatid parasites has been identified in honeybees^21,22^. According to recently published phylogenomic data, these parasites would cluster together with the genus *Leptomonas* and other *Crithidia* species forming the sub-family Crithidiatae^23^. Among the species infecting honeybees, *L. passim* is the most prevalent, having been found in the 15–60% of the colonies worldwide, reflecting the wide range of ecological scenarios where these parasites thrive and transmit^24–30^. This parasite has been associated with winter colony losses^30^ and recent data have demonstrated the reduction of the life span of honeybees experimentally infected with *L. passim*^31^.

As a result of the characterization of honeybee-infective trypanosomatid parasites cell cultures, we have found that these protozoan microorganisms can form biofilm micro-colonies both in vitro and in vivo. These multicellular stages are generated via a sequential process involving the secretion of promastigote EPS and the further transformation of the long-flagellated promastigote form into multicellular haptomonad biofilms (HB) matrixes that proliferate in the hindgut of honeybees. We also characterized the structure and proteome of the EPSs and demonstrated the higher survival of *L. passim* and *C. mellificae* monoxenous parasites to cold-shock and hypoosmotic stress compared with other monoxenous and dixenous trypanosomatid parasites. This data demonstrates that secretion of EPS, and further transformation into trypanosomatid biofilms, is a widespread strategy used by these parasites for survival, resilience, and dispersal in honeybee hosts.

## Results

### *Lotmaria passim* promastigote forms secrete EPS

**Fig. 1:**
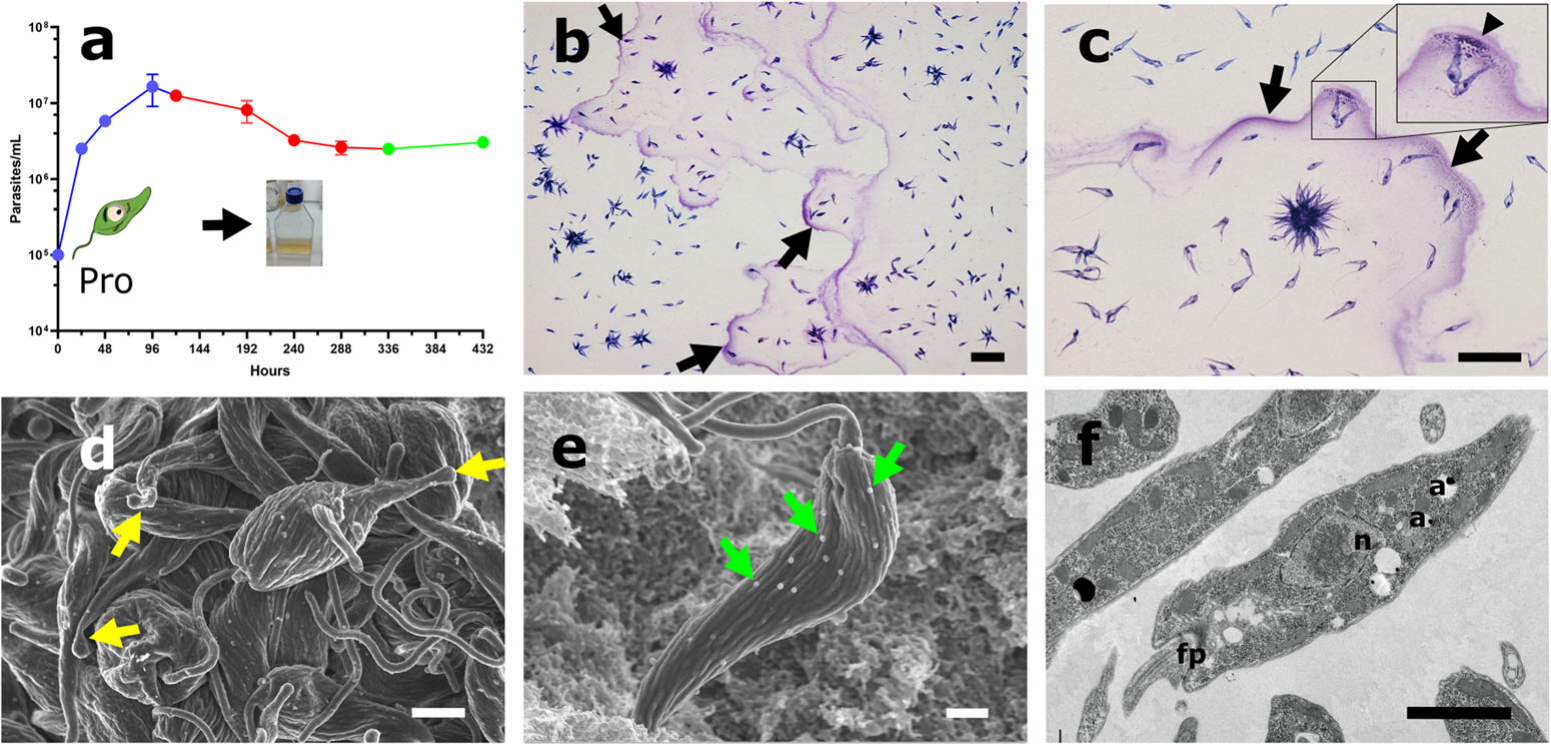
*Lotmaria passim* promastigote morphology, growth, and EPS secretion during the log phase of growth. **a** Growth curve of *L. passim* parasites. Promastigotes (Pro) growth at log phase is shown in blue. The flask represents a culture at logarithmic phase of growth. Early haptomonad-like biofilm transformation at stationary phase is shown in red and late haptomonad-like biofilms in green. Curves are mean ± s.d. **b**, **c** Giemsa stain of promastigotes at log phase of growth. Note the large fibers of polymerized EPS (black arrows) and vesicles (black arrow heads). Scale bar represents 25 µm. **d**, **e** Scanning Electron Microscopy of log phase promastigotes. Yellow arrows indicates “nozzle” shape phenotype in many promastigotes. Scale bars represents 1 and 2 µm respectively. **f** Transmission Electron Microscopy showing a prototypical morphology of a promastigote. Note the single flagellum protruding from the flagellar pocket (fp), the nucleus (n) and acidocalcisomes (**a**). Scale bar represents 2 µm.

To seek for developmental changes and to characterize the growth curve of *L. passim* C3 strain in vitro, we performed a microscopic analysis with a low passage cell cultures (passage 1) from frozen stocks at passage 0 of freshly isolated parasites. The parasites were analyzed at both logarithmic (earlylog, midlog and latelog) and stationary phases of the growth curve. During the logarithmic phase, *L. passim* showed free-swimming promastigote forms eventually arranged in rosettes, with rapid motility and a doubling time (D.T) of 9.3 ± 0.3 h (Fig. 1a–c). To our surprise, we noticed the accumulative presence of polymeric substances forming long fibers (Fig. 1b, c) during the midlog and latelog phases of growth defined hereafter as promastigote EPS. The morphological and ultrastructural analysis of promastigotes by TEM and SEM showed a promastigote prototypical structure with an elongated flagellum with a clear “nozzle” phenotype towards the apical end of the cell body (Fig. 1d), the presence of numerous extracellular vesicles released from the cell surface (Fig. 1e) and a flagellar pocket at the anterior end of the cell with acidocalcisomes spread throughout the cytoplasm (Fig. 1f).

**Fig. 2:**
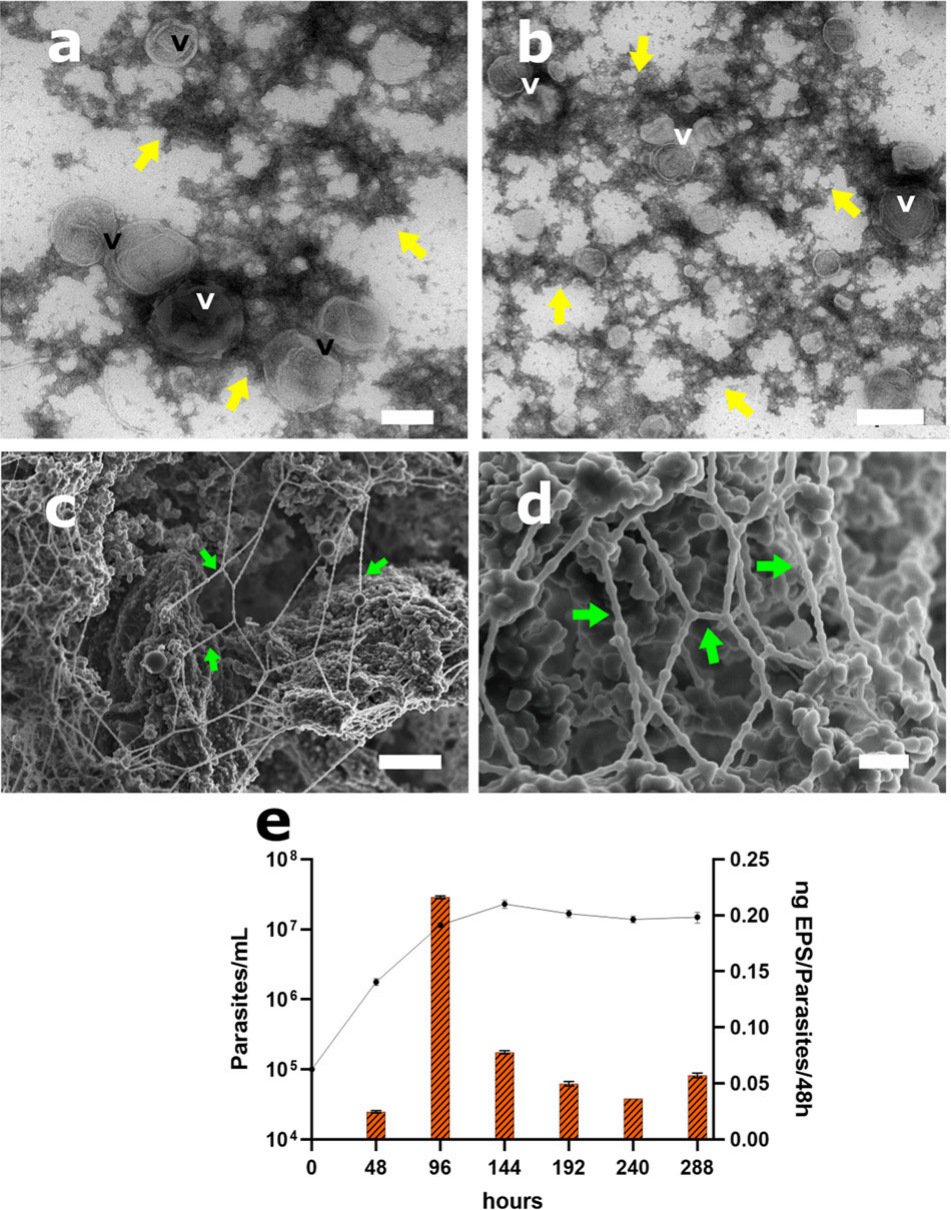
Structure and secretion kinetics of *L. passim* Extracellular Polymeric Substances (EPS). **a**, **b** Transmission Electron Microscopy of EPS. Note the amorphous and lumpy appearance of the biopolymer (yellow arrows) and the presence of EVs intercalated across the structure forming spherulites. Scale bars represents 200 and 500 nm respectively. **c**, **d** Scanning Electron Microscopy of the EPS. A detail of the dense EPS mass obtained from *L. passim* cell cultures as well as the long fibers composed by repetitive monomeric structures (spherulites, green arrows) bound forming EPS meshes. Scale bars represents 1 µm and 200 nm respectively. **e** Time-lapse quantification of EPS secretion measured as EPS dry-weight during growth curve of *L. passim*. The lines in graph stated for the growth curve and the bars stated for the yields of secreted EPS measured in nanograms of EPS secreted each 48 h and presented as mean ± s.d. Note that the peak of secretion is located at the late log phase of growth at approximately 1 × 10^7^ cells/ml.

Transmission electron microscopy (TEM) analysis of the purified EPS showed numerous extracellular vesicles (EVs) associated with electron dense fiber-like structures of the EPS defined hereafter as spherulites (Fig. 2a, b). As revealed by Scanning Electron Microscopy (SEM), the basic units of promastigote EPS were repetitive spherulitic structures formed by different monomers of different nanometer size (Fig. 2c, d, and Supplementary Fig. 1). The analysis of particle size of the EPS revealed a wide range of diameters from 50 to 390 nm with a median size of 149.6 nm (Supplementary Fig. 1). These repeating units were organized either as collapsed masses or long fiber chains. Intriguingly, the long fibers showed 3D structures resembling “rosary beads” that eventually polymerized like EPS meshes. It is worth noting that long fibers showed a spatial repetitive pattern of organization with round-shaped spherulitic material which was bound with constant repetitive distances (Fig. 2c, d and Supplementary Fig. 2).

To analyze the EPS release dynamics of *L. passim* parasites, we quantified the secretion of the biopolymer every 48 h during the growth curve from 0 to 336 h post-inoculum. The parasites showed the maximum EPS formation at 1×10^7^ cells/mL (96 h post-inoculum) coincident with the latelog phase of growth (no biofilm layer observed), with a calculated yield of 0.2 ng EPS/parasite/48 h (Fig. 2e). This time point of maximum EPS formation was chosen for purification and further morphological, structural and proteomic analysis of the secreted EPS.

Once the secretion dynamics and structural architecture of the EPS were defined, we performed a proteomic analysis of *L. passim* EPS to analyze the composition of such biopolymer using previously reported protocol for extracellular vesicle isolation in other trypanosomatids^32,33^. As a result, 197 proteins were identified in the EPS proteome in at least two out of three biological replicates. In order to analyze the differences between monoxenous and dixenous trypanosomatid parasites, the proteins found in the EPS fraction of *L. passim* were compared with proteins found in EVs of *L. major*^33^ and *T. cruzi*^32^ insect forms that were also obtained using differential centrifugation. The analysis showed a core of 36 proteins present in the three proteomes and, additionally, 59 and 30 proteins shared with *L. major* and *T. cruzi* respectively. A total of 58 proteins were specific of the *L. passim* EPS proteome (Supplementary Fig. 3a). The Gene Ontology (GO) enrichments of biological processes and molecular functions revealed an enrichment in unfolded protein binding, protein folding and vacuole as the most representative GO term enrichments in the *L. passim* EPS (Supplementary Fig. 3b). The EPS proteome was also analyzed for Metabolic Pathway Enrichments. The results showed an enrichment of ATP biosynthetic pathway, different steps of the glycolysis as well as processes related with anabolic and catabolic processing of sugars, such as sucrose biosynthesis and different steps of gluconeogenesis (Supplementary Fig. 3c).

### *Lotmaria passim* promastigote forms are capable of differentiating into haptomonad-like biofilms in vitro

**Fig. 3:**
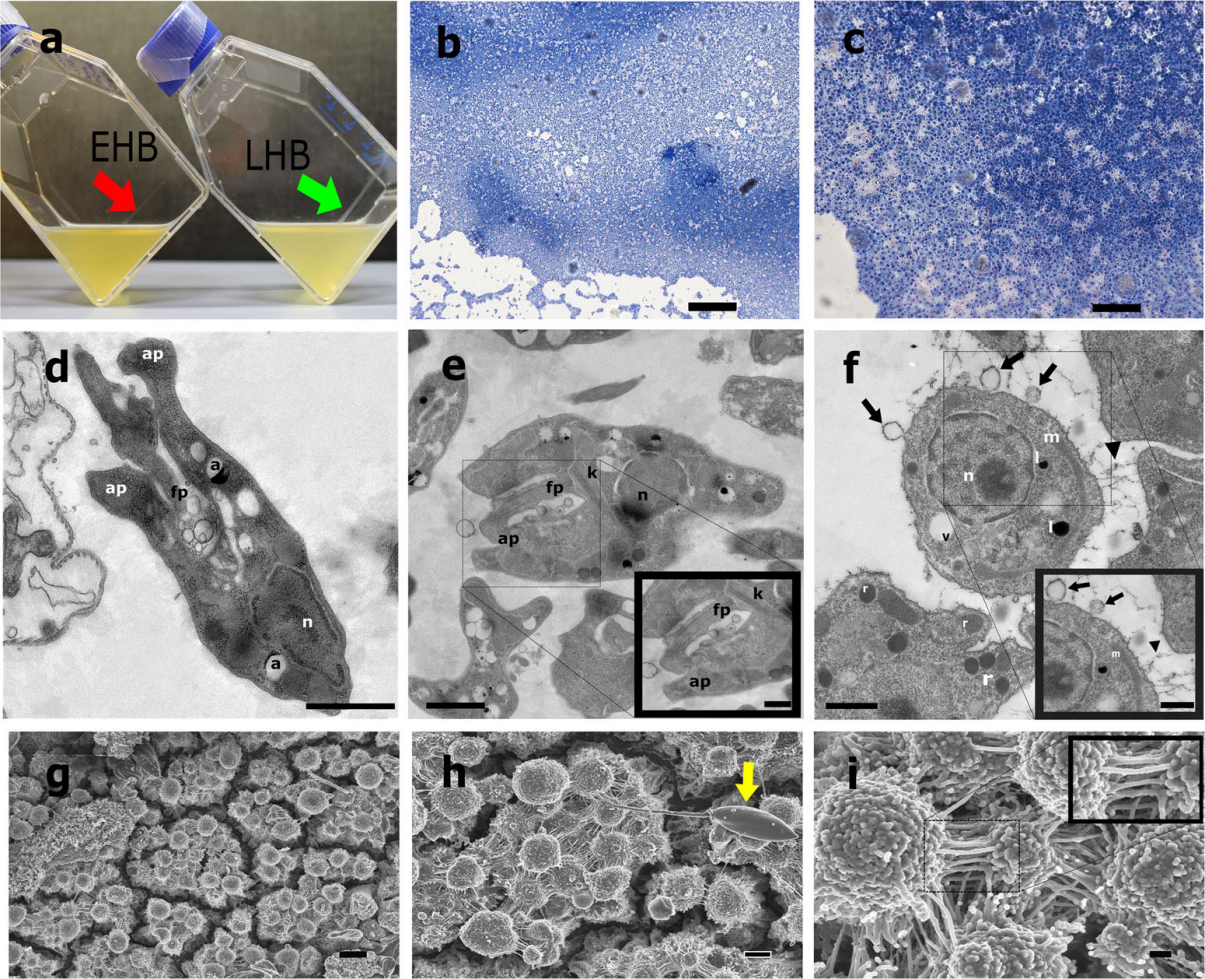
*Lotmaria passim* differentiation into haptomonad-like biofilms in vitro. **a** Image of cell culture flasks at stationary phase 7 days post inoculation (early haptomonad-like biofilms (EHB)) and 20 days post-inoculation (late haptomonad-like biofilms (LHB)). Note the increase in thickness of the layer of haptomonad biofilms at the air-liquid interphase. **b**, **c** Giemsa stain of haptomonad-like biofilms adhered to cell culture flasks. Even after the extensive washing of those flasks for Giemsa stain, numerous haptomonad cells remain tightly bound indicating the strong attachment of those cells. Scale bars represents 100 µm and 25 µm respectively **d**, **e** Detached haptomonad forms analyzed by Transmission Electron Microscopy. Note the reduction in flagellar length within an enlarged flagellar pocket (fp) close to the kinetoplast (k). The bulge at the base of the oval-shaped cell body and the attachment pads (ap) are also indicated. Scale bars represents 1 µm. **f** Transmission Electron Microscopy of transverse sections of haptomonad-like biofilms. Note the release of spherulites (black arrows) and the polymeric fibers binding contiguous cells of a biofilm colony (black arrow heads). Scale bar represents 1 µm. **g**–**i** Haptomonad-like biofilms analyzed by Scanning Electron Microscopy. Note the separation of microcolonies by channels, the presence of a promastigote without EPS in its surface (yellow arrows) and the tight interconnection of the cells with long fibers formed by spherulites. Scale bars represents 4 µm, 2 µm and 400 nm. The following abbreviations are used in the figures: Acidocalcisomes (a), flagellar pocket (fp), attachment pads (ap), nucleus (n), kinetoplast (k), mitochondria (m), vacuole (v), extracellular polymeric substances (black arrow heads).

To analyze whether or not *L. passim* was capable to differentiate into sessile biofilm multicellular lifestyle, we characterized the stationary phase of *L. passim* cell cultures. Approximately 24 h after the maximum yield of promastigote EPS secretion at the latelog phase, parasites showed a spontaneous developmental differentiation towards surface-attached haptomonad-like forms at the air/liquid interphase (Fig. 3a) defined hereafter as haptomonad-like biofilms (HB). The HB showed a continuous growth from a thin layer to a thicker layer (Fig. 3a) that we called as early haptomonad-like biofilms (EHB, 7 days of culture), and late haptomonad-like biofilms (LHB, 20 days of culture).

The morphological and ultrastructural analysis of the HB (Fig. 3b–i and Supplementary Fig. 4) showed a classical haptomonad morphotype with an oval shape and reduced length of the cell body compared to promastigote forms. The cells were surface-attached, which would influence the redifferentiation of the cell body basement and flagella to form an attachment plaque as it was previously described for another trypanosomatid species^14^ (Fig. 3d, e). Transformation into EHB and further LHB was accompanied by an accumulation of a dense EPS coating with projections connecting cells which would serve as a scaffold to increase the internal stability of the multicellular aggregate (Fig. 3f–i and Supplementary Fig. 4). This electron-dense biopolymeric matrix was also formed by repetitive round-shaped spherulitic material that were distributed among and also emerging from the cells (Fig. 3f and Supplementary Fig. 4). The SEM analysis showed that LHB are structured by independent micro-colonies separated by connected channels and funnels which would delimitate and communicate every foundational cluster of cells (Fig. 3g–i). Besides, we observed the continuous presence of the promastigote forms eventually distributed over the biofilm and actively secreting EVs in the liquid media during the stationary phase (Fig. 3g, h and Supplementary Fig. 4). Of note, promastigote forms actively release EVs but were not covered by the EPS matrix.

The capacity to secrete EPS by free-swimming unicellular stages and to form fully formed biofilms at stationary phase was also confirmed in other monoxenous trypanosomatid parasites of honeybees such as *C. mellificae* (Supplementary Fig. 5) that showed a D.T of 4.4 h ± 0.1 h. Despite the differences in morphology and growth rates, these results demonstrated that different monoxenous trypanosomatid parasite species of honeybees have developed similar evolutionary strategies for adhesion and colonization of surfaces.

### *Lotmaria passim* haptomonad biofilms are attached to the hindgut of honeybees while promastigote forms are released by feces

**Fig. 4:**
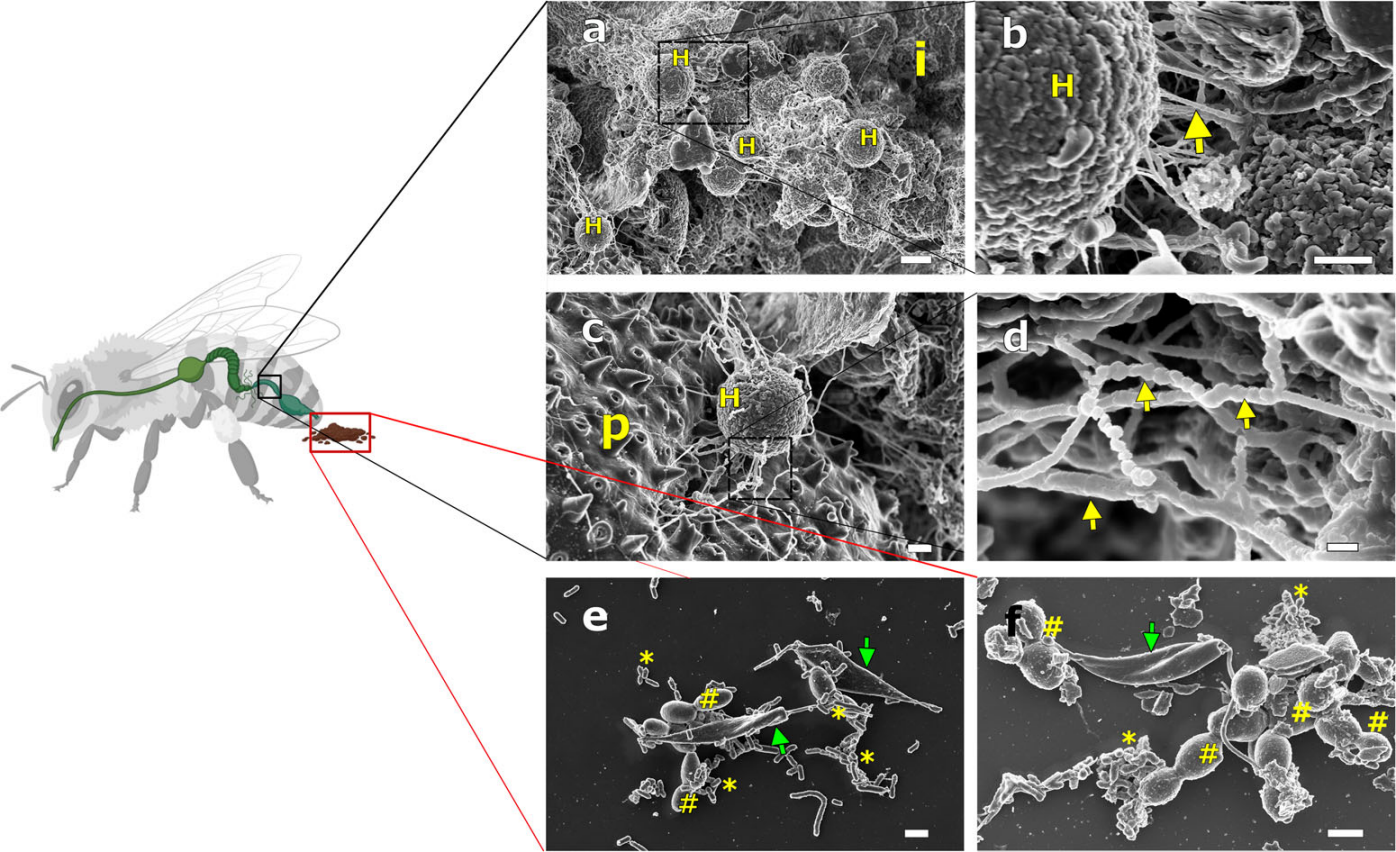
Scanning Electron Microscopy analysis of *L. passim* life cycle stages in experimental infections of honeybees. **a**, **b** Haptomonad biofilm colonies composed by several haptomonad cells attached by multiple EPS fibers. Scale bars represents 3 µm and 1 µm. **c** An haptomonad cell attached to a pollen grain (p) through multiple EPS projections in the ileon of an infected honeybee. Scale bar represents 1 µm. **d** A closer image of an EPS formed in vivo by *L. passim* composed by monomeric spherulites forming a “rosary bead” characteristic structure (yellow arrow). Haptomonad (H), ileum (i), pollen (p). Scale bar represents 200 nm. **e**, **f** Fecal content of *L. passim* infected honeybees. Scale bar represents 2 µm. Note the presence of flagellated planktonic promastigote forms (green arrows) surrounded by bacteria (*) and yeast cells (#).

To ascertain whether or not trypanosomatid biofilms were also formed in vivo, experimental infections of honeybees with *L. passim* promastigote forms were performed. The SEM analysis showed the formation of fully mature haptomonad biofilms in the honeybee hindgut (Fig. 4a–d, Supplementary Figs. 6 and 7). Likewise, we also found the presence of spherulitic material forming dense and long fibers interconnecting the cell clusters (Fig. 4d and Supplementary Fig. 6a–c), indicating that ligation and cementation of those surface-attached colonies follow a canonical pathway similarly to what was found in vitro. However, the analysis of the parasite forms present in feces did not result in the localization of any individual or multicellular haptomonad biofilm. The feces showed the abundant presence of promastigote forms (3.1 ×10^4^ ± 2.1 ×10^4^ cells per fecal sample between days 5 and 7 post-infection) bound to clusters of bacteria and yeast cells (Fig. 4e, f and Supplementary Fig. 6d–f). As observed in vitro, promastigote forms were highly motile (data not shown) with a continuous secretion of EVs to the extracellular milieu (Fig. 4e, f). This result suggests that haptomonad biofilm is a stage that facilitates *L. passim* adhesion and thriving within the bee-hindgut, being the promastigote form reluctant to hindgut adhesion and thus released by feces.

### *Lotmaria passim* haptomonad-like forms showed an increased cell death but also higher capacity of sugar degradation and antioxidant activity

**Fig. 5:**
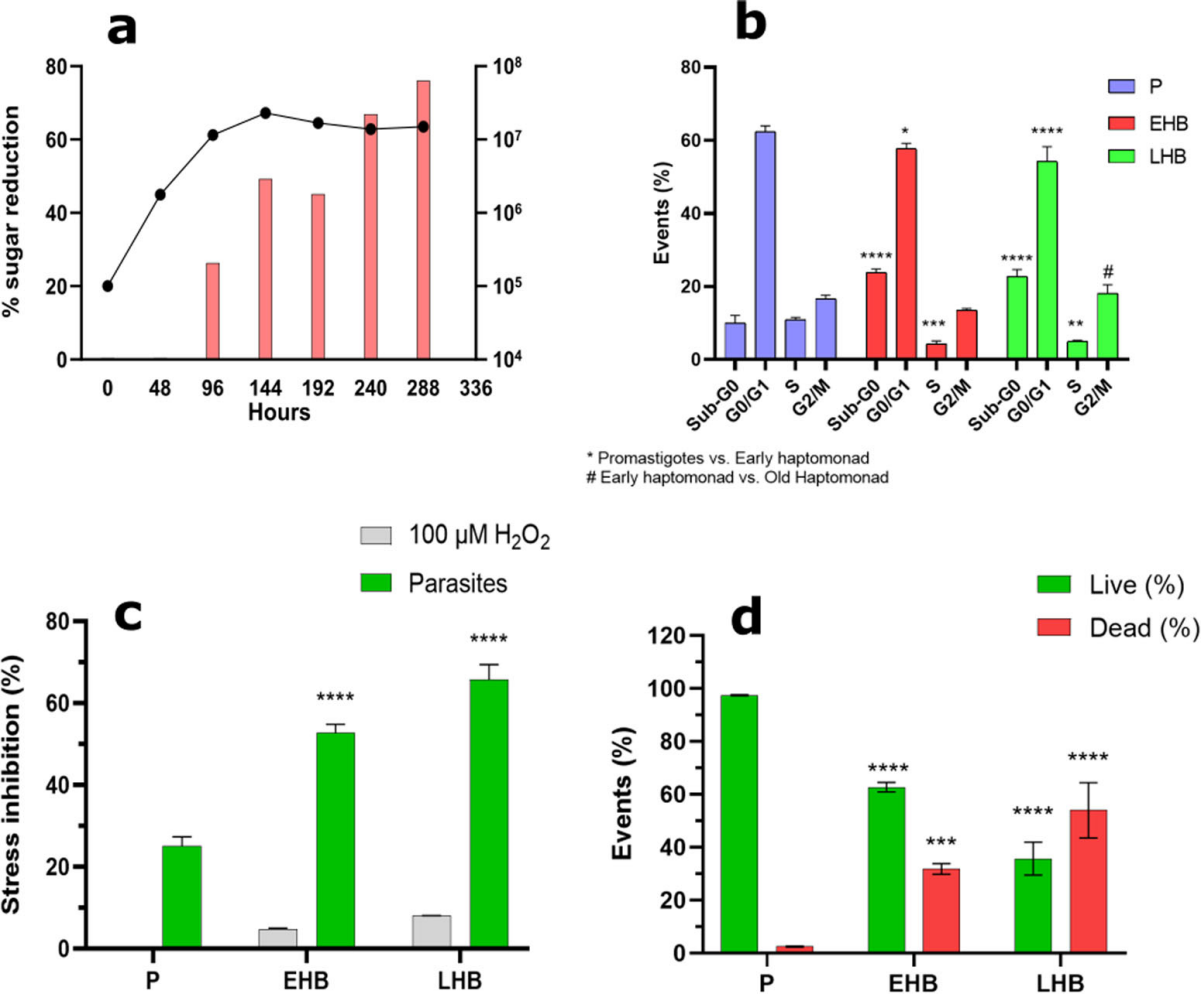
Differential biochemical and cellular properties of promastigotes, early haptomonad-like biofilms and late haptomonad-like biofilms in *L. passim* in vitro. **a** Sugar reduction percentage of cell culture supernatants during in vitro cell growth curve. The bar graphs indicates the increase in reduced sugars towards the stationary phase. **b** Cell cycle analysis using propidium iodide and flow cytometry of the different life cycle forms of *L. passim*. The y-axis indicates the percentage of events for each cell cycle phase. **c** Percentage of oxidative stress inhibition in *L. passim* life cycle stages labeled with H_2_DCFDA to evaluate the redox levels in the different life cycle forms of *L. passim*. 100 μM hydrogen peroxide (H_2_O_2_). was used as positive control. **d** Determination of cell viability by flow cytometry in the different parasite forms using 5 μM Syto9 (green fluorescence, live cells) and 40 μg/mL PI (red fluorescence, dead cells). Data is presented as mean ± s.d. For statistical comparisons two-way ANOVA followed by Tukey´s post hoc test was performed. ****P ≤ 0.0001; ***P ≤ 0.0004; **P ≤ 0.001; *P ≤ 0.01.

To search for differences among the three *L. passim* life cycle stages (Promastigote, EHB and LHB), different biochemical parameters were employed. We evaluated reducing sugars in the culture media to check for the capacity of degrading polysaccharides such as promastigote EPS, cell cycle to check for DNA degradation and duplication, antioxidant capacity to evaluate which parasite form show higher resistance to Reactive Oxygen Species and also the proportions of live/dead cells in the cell cultures since cell death is an intrinsic property of biofilms^34^. The time-lapse analysis showed that EHB and LHB were more active than promastigotes in generating reduced sugars, increasing this activity from 30 to 75% throughout the growth of the biofilms at the stationary phase (Fig. 5a). The cell cycle analysis showed that the S phase in EHB and LHB was significantly reduced with respect to promastigote forms (5.3% and 4. 9% vs 10% of the cells) whereas G2/M was maintained (Fig. 5b). Noteworthy, the sub G0 stage cell population was significantly elevated in EHB and LHB indicating an increase in apoptotic cells and DNA degradation (Fig. 5b and Supplementary Fig. 8). The analysis of the oxidative stress measured with H_2_DCFDA of the three parasite forms showed the highest levels of inhibition in LHB and EHB compared with promastigote forms (65.8% and 52. 7% vs 25.1% stress inhibition capacity) (Fig. 5c and Supplementary Fig. 8). To evaluate the live/dead ratio of the biofilm, EHB and LHB were labeled with Syto9 and PI, and compared with the viability of promastigote forms as control. The percentage of cells alive was of 97.4% in promastigotes, 62.7% in EHB and 35.7% in LHB, which would demonstrate that fully mature biofilms are composed by an increasing number of dead cells (Fig. 5d and Supplementary Fig. 9). Therefore, haptomonad-like biofilms are composed by replicative living cells and non-replicative dead cells similarly to what has been found in other microorganisms^1,2,5^, with an increased capacity of producing reducing sugars and resistance to oxidative stress.

### Flagellated *L. passim* promastigote and *C. mellificae* choanomastigote forms are highly resistant to cold-shock and hypoosmotic stress

**Fig. 6:**
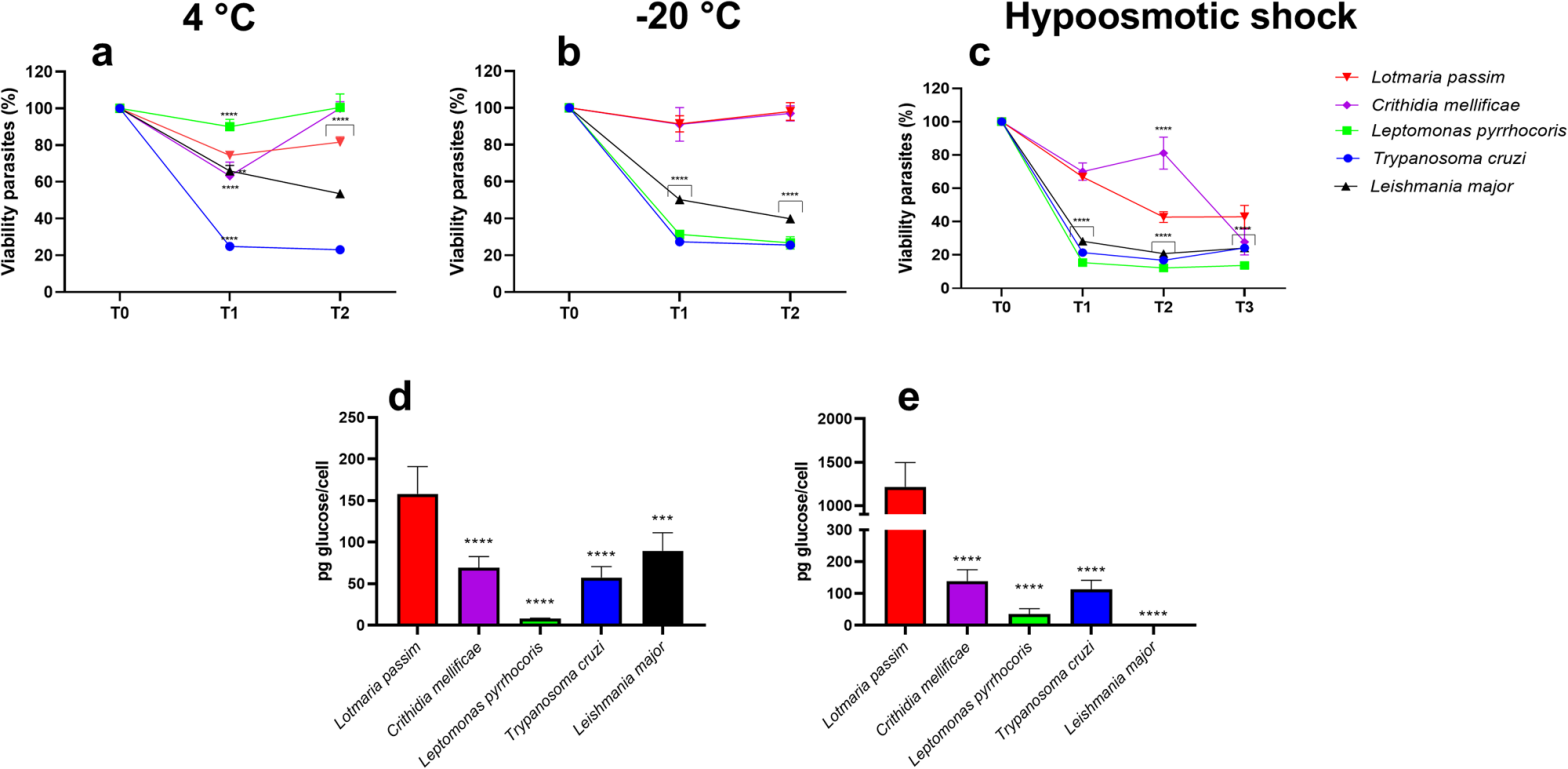
Stress resistance and sugar secretion of monoxenous and dixenous trypanosomatid parasites. **a**, **b** Viability of planktonic forms at 24 h (T1) and 72 h (T2) after cold-shock treatments at 4 °C, and −20 °C. The percentage of viability was obtained by comparing with non-treated cells (T0). **c** Viability of planktonic forms at 24 h (T1) and 72 h (T2) and 144 h (T3) after hypoosmotic shock. The viability of the parasites after treatments was measured spectrophotometrically using resazurin and the percentage of metabolic reduction and transformation into resofurin compared with non-treated cells (T0). Quantification of the polysaccharides presents in cell pellets (**d**) and supernatant (**e**) fractions in the late log phase of *L. passim* promastigotes, *Crithidia mellificae* choanomastigotes, *Leptomonas pyrrochoris* promastigotes, *Trypanosoma cruzi* epimastigotes and *Leishmania major* promastigotes. The measurements were normalized to picograms of glucose per cell. Data is presented as mean ± s.d. For statistical comparisons Two-way ANOVA followed by Tukey´s post hoc test was performed. ****P ≤ 0.0001; ***P ≤ 0.0004, **P ≤ 0.001; *P ≤ 0.01.

To ascertain whether or not monoxenous trypanosomatid parasites of honeybees have generated any functional gain to overcome changes in their environmental milieu, we performed a time-lapse experiment to analyze the viability of different species of monoxenous and dixenous trypanosomatid parasites under cold-shock and hypoosmotic stress. We selected hypoosmotic conditions since parasites must be exposed to changes in osmolarity depending on the host diet, location in the gut and once released by feces. Different cold-shock temperatures (4 °C, −20 °C) were chosen based on previous observations after thawing *L. passim* cultures, and due to the possible exposure of the parasites to low temperatures, once released by feces. Firstly, the performance of flagellated-insect forms of *L. passim*, *C. mellificae*, versus promastigote insect stage of the monoxenous parasite of firebugs *L. pyrrochoris* (Supplementary Fig. 10) and the human-infective dixenous parasites *L. major* promastigotes and *T. cruzi* epimastigotes was analyzed. Since *L. pyrrochoris, L. major* and *T. cruzi* did not show any EPS secretion in their cultures, those parasites were used as non-EPS secreting controls. Overall, monoxenous trypanosomatids of honeybees showed the highest resistance to cold-shock with viabilities ranging 100–75% for the promastigotes forms of *L. passim* and 100–63% for the choanomastigote forms of *C. mellificae*. By its hand, *L. pyrrochoris* showed a high performance at 4 °C (viabilities between 100–90%) falling down to 25% at freezing conditions. Finally, *T. cruzi* epimastigotes and *L. major* promastigotes showed the lowest viabilities, with viabilities between 23–27% and 67–39%, respectively (Fig. 6a, b). When the same trypanosomatid forms were subjected to hypoosmotic shock, again *L. passim* promastigotes and *C. mellificae* choanomastigotes showed the highest viabilities of 66–32% and 81–27%, respectively. All the other species tested showed a marked decrease in their viabilities, with values between 15–11% for *L. pyrrochoris*, 21–16% for *T. cruzi* and 27–20% for *L. major* (Fig. 6c). Overall and with the exception of the viability showed by *L. pyrrochochris* at 4 °C, the flagellated unicellular forms of monoxenous trypanosomatid parasites of honeybees showed the highest resistance in all timings and conditions tested.

Next, we have also compared the difference in cell viability of flagellated forms versus EHB and LHB forms of *L. passim* and *C. mellificae* under the same stress conditions. Again, the promastigote and choanomastigote forms resulted in the highest resistance to cold shock and hypoosmotic stress at 24 h post-treatment. In the case of hypoosmotic stress, EHB and LHB recovered from this stress increasing their viabilities over promastigotes and choanomastigotes at 144 h post-treatment (Supplementary Fig. 11).

Since EPS are mainly composed of polysaccharides, we corroborated the differences in sugar content in the supernatant (loosely bound) and cell pellet (tightly bound) fractions of *L. passim* promastigotes, *C. mellificae* choanomastigotes, *L. pyrrochoris* promastigotes, *T. cruzi* epimastigotes and *L. major* promastigotes. In both fractions, *L. passim* showed significant higher sugar concentration (Fig. 6d, e), with and special increase in cell culture supernatants where this parasite shows >8-fold increase in total sugar content compared with the other trypanosomatid species assayed (Fig. 6d). Overall, this data strongly supports that *L. passim* might have an increased resistance to environmental stress due to the secretion of EPSs composed by tightly and loosely bound polysaccharide matrices.

## Discussion

Monoxenous trypanosomatid parasites are a wide extended group of organisms with an intrinsic diversity of forms that have successfully colonized a myriad of insect species, including honeybees^7^, and whose mechanisms for transmission and survival within and outside their hosts are still poorly understood. During the course of the characterization of the life cycle of *L. passim* in honeybees, we have found that the parasites were capable to differentiate into multicellular biofilms communities. The developmental program towards biofilms occurs in a stepwise process involving a heavy secretion of promastigote EPS at late log phase of growth, followed by a process of soluble cell aggregation and formation of surface-attached haptomonad biofilms microcolonies at the air-liquid interphase of in vitro static cell cultures. Moreover, this developmental differentiation program was also found in experimental infections of honeybees with *L. passim*.

This work demonstrated that *L. passim* is able to secrete EPS, a constitutive component of haptomonad biofilms. The ubiquity of haptomonad biofilms in *L. passim* cell cultures and the honeybee hindgut points towards the essentiality of this developmental process for the successful colonization of *L. passim* within their host. Moreover, we also found how other monoxenous trypanosomatid parasites such as *C. mellificae* are also capable of differentiating into biofilm lifestyle, indicating that this strategy is employed by different trypanosomatid protozoan cells in honeybees. These results would explain the dense mass of filaments previously found surrounding the haptomonad stage of *L. passim, C. mellificae*, or *C. acantocephali* in infected honeybees^15,35^. However, and despite the architectural commonalities, the molecular and biochemical similarities between in vitro and in vivo haptomonad biofilms have not been approached in this work, which would be necessary to study in future research. Here, the microscopic analysis of EPS matrixes in vitro and in vivo revealed the formation of fiber chains composed by repetitive monomeric units that we have called as EPS spherulites. In this process, promastigotes would play an essential role providing the scaffold material for the polymerization of EPS filaments since this forms show a promiscuous secretion of EVs and polysaccharidic material to the extracellular milieu. Thus, free-swimming unicellular *L. passim* promastigotes would be the main EPS-producer forms, which is in agreement with the canonical model of biofilm formation found in bacteria or fungi^1,2,5^. Nevertheless, it cannot be ruled out the possible heterogenic nature of the EPS in vivo since EPS-producing bacteria such as *Lactobacillus* spp. or *Snodgrassella alvi* are also present in the bee hindgut^36^.

The proteomic comparison between secretomes of dixenous and monoxenous parasites showed that 58 out of 244 proteins were specifically found in *L. passim* EPS. Interestingly, several of these proteins were previously described as part of the EPS biosynthetic machinery in other organisms. For instance, we found the enzyme Beta-fructofuranosidase that have been previously described with a dual role in the hydrolysis of sucrose or in EPS polymerization in *Bacillus* sp^37^ or the helicase RuvB, which would destabilize the lattice-like structures formed by the eDNA present in the EPS of different species of bacteria^38,39^. The EPS proteome also contain examples of enzymes such as a flavin reductase and spermidine synthase, which in turn are implicated in the processing of riboflavin and spermidine and identified as a part of the quorum sensing system for inducing biofilm formation in different bacterial species^40–42^.

The secretion of this biopolymer might represent a metabolic cost for honeybee trypanosomatid parasites that might slow down the growth rates of parasites. This may be supported by the higher total sugar content (8 fold higher in cell culture supernatants) and slower doubling time (9.3. h vs 4.4 h) found in *L. passim* promastigotes compared to *C. mellificae*. Nevetherless, the duplication rates of those two honeybee-infecting and EPS-secreting species are among other monoxenous or dixenous trypanosomatid such as *L. pyrrochoris* promastigotes (D.T: 4.2 h)^43^, *L. mexicana* promastigote forms (D.T: 7.1 h)^44^ or *T. cruzi* epimastigote forms (D-T: 36.24 h)^45^.

We also found that *L. passim* biofilms have an increased resistance to oxidative stress. The release of hosts ROSs is a major component of insect innate immune pathways regulating gut-microbe homeostasis and mediating pathogen killing. Indeed, this defense mechanism was found to be upregulated in *Anopheles* mosquitoes infected with *Plasmodium vivax*^46^ or in infections of sandflies with *Leishmania mexicana*^47^. In this context, the higher resistance to ROS of *L. passim* haptomonad biofilms would be a selective advantage for evading the innate immunity of the honeybee. Moreover, the comparison with respect the insect-replicating stages of dixenous parasites resulted in a higher resistance of the three *L. passim* forms assayed (promastigotes, EHB and LHB) to both cold-shock and osmotic stress and where the promastigote forms showed the higher levels of resistance to cold-shock. Despite biofilms are commonly defined as resistant and recalcitrant to numerous types of stress, also free-swimming unicellular stages of different bacteria could show and elevated resistance to hypoosmotic conditions as demonstrated for *Enteroccocus faecalis* and *Streptococcus sanguinis* present in the root canal system in clinical endodontics^48^, or in the saprophytic soil bacterium *Pseudomonas putida*, where this resistance is even higher than the biofilm forming counterpart species^49^. Given that *L. passim* promastigotes are the main EPS producers, this secretion may supply to the parasites of osmotically-active solutes that would act balancing the osmotic and temperature changes in the surrounding media. Indeed, EPS composition could be modulated in response to extreme environmental conditions, such as in the antartic marine bacteria where this secretion act as cryoprotectant by increasing galactose content in the polysaccharide matrix^50^. Since, promastigotes were the only parasite stage found in honeybee feces, we propose that these forms would potentially be adapted to rapid changes in environmental pressures outside their hosts. Further work characterizing EPS sugar composition would be necessary for the understanding of the protective effects observed in EPS-secreting trypanosomatid parasites.

**Fig. 7:**
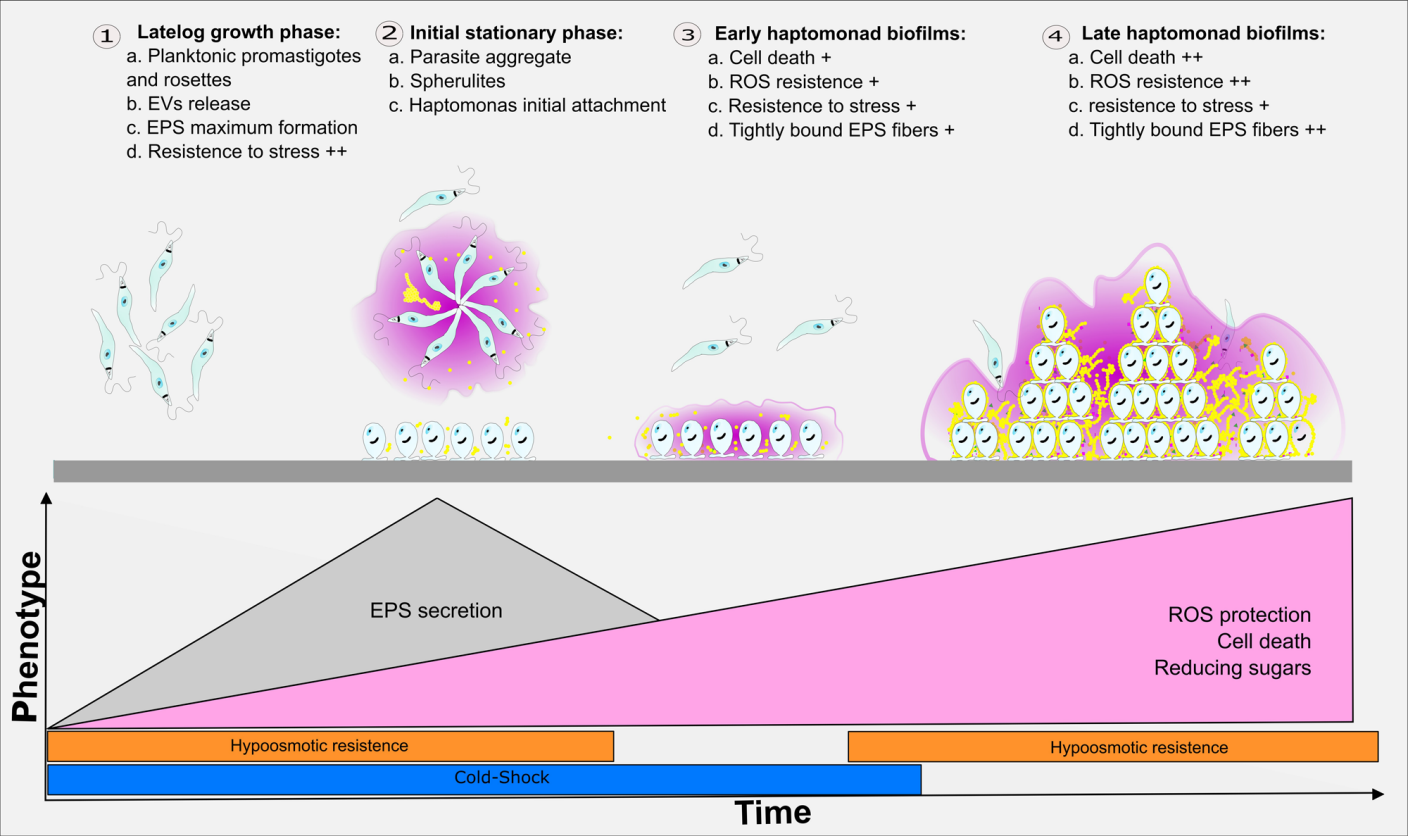
In vitro model of *L. passim* differentiation into biofilms. (1) The promastigote forms during the logarithmic phase of growth (1 × 10^7^ cells/ml) have the maximum capacity of EPS formation (shown in purple) and resistance to hypoosmotic and cold shock stress. (2) Once stationary phase is reached, the parasites start to attach to the cell flask surface at the air/liquid interphase, and differentiate into haptomonad-like forms. (3) Early-haptomonad biofilms have not fully formed EPS matrix, and are characterized by and increasing percentage of cell death and resistance to Reactive Oxigen Species (ROS). (4) After up to 20 days haptomonad-like biofilms are formed by a multitude of microcolonies where cells are tightly bound through EPS fibers. Late-haptomonad biofilms have the maximum levels of ROS resistance, sugar reduction and dead cells in the polymeric matrix. Late-haptomonad biofilms are also found in the honeybee hindgut.

Overall, this work describes the secretion of EPS by *L. passim* promastigotes and developmental differentiation into trypanosomatid biofilms (resumed in Fig. 7). These results show the need for further studies to elucidate the molecular mechanisms behind the release and formation of trypanosomatid EPS and biofilms and thus towards the understanding of the transmission and resilience of these parasites in honeybees.

## Methods

### Trypanosomatid cultures and EPS purification

The strain used for the present study and named *L. passim* C3, was isolated from an infected honeybee colony of an apiary located in Barreras, Cortijo Tinajas (Beznar, Granada 36°55'25.1“N, 3°31'42.9“W. Primary cultures of this strain were generated and cultured in modified BHT medium as previously described^51^ and details provided at NCBI as BioSample: SAMN30034964. *Crithidia mellificae* choanomastigotes and *L. pyrrochoris* promastigotes were also cultured in modified BHT medium. The later was isolated by our team in July 2023 from *Pyrrochoris apterus* near the Faculty of Sciences of the University of Granada 37°10'54.3“N, 3°36'37.1“W and was called as *L. pyrrochoris* C1 strain. To verify the species, gDNA was purified from axenic cell cultures and sequenced sanger sequencing of 18S rRNA using the primers S763 and S762 from Maslov et al.^47^. A Giemsa stain of *L. pyrrochoris* C1 strain was shown in Supplementary Fig. 10.

For the comparative study with dixenous trypanosomatid parasites, *L*. *major* Friedlin strain was cultured in RPMI 1640 medium (pH 7.2; Thermo Fisher Scientific), 2 mM glutamine (Merck) and 10% iFBS (Thermo Fisher Scientific) and *T. cruzi* Y strain in Liver Infusion Tryptose (LIT) medium (pH 7.4) supplemented with 10% iFBS^52^.

Haptomonad-like biofilms were obtained at the stationary phase of cultures as a visible layer at the liquid/air interphase of culture in static conditions (starting) at 28 °C. The biofilms were recovered at day 7 (early haptomonad-like biofilms (EHB)) and 20 (late haptomonad-like biofilms (LHB)) after the initial inoculum of 1 × 10^5^ cells/ml. According to previous experimental infection assays, these two-time points represent the moment before and after an increased mortality of bees^51^. To isolate the biofilms, the cultures were washed 3 times in phosphate saline buffer (PBS) 0.1 M pH 7.4 and the biofilm layer scraped and detached from cell flasks using a rubber policeman (Avantor).

To isolate EPS from cell cultures, promastigote forms at late logarithmic growth phase (1 × 10^7^cels/ml) were centrifuged at 2000 × *g* and the supernatant (soluble EPS) precipitated with 1 volume of chilled (−20 °C) 100% ethanol overnight. Next, the supernatants were centrifuged at 4000 × *g* for 30 min. The EPS pellets were dialyzed overnight (dialysis membrane MWCO 12–14 kDa (Thermo Fisher Scientific) to eliminate free monosaccharides and salts sugars from culture media. Finally, the dialyzed EPS were precipitated again in Ethanol 100% as before, centrifuged at 4000 × *g* and the pellets stored at −20 °C until further processing.

### EPS quantification

The soluble EPS were quantified by total dry weight determination and sulfuric-acid method for measuring the polysaccharidic fraction^53^. To do the later, a standard curve with glucose was generated to quantify the purified EPS. Next, 200 μl of the samples were mixed with 200 μl phenol (5%) in glass tubes. Then, 1 mL of sulfuric acid concentrated (98% w/v) was added and incubated for 10 min on ice. Finally, a volume of 100 μl of the reaction was transferred to 96-well plates and read in a Multiskan® Spectrum spectrophotometer (Thermo Fisher Scientific) at 490 nm.

### Determination of sugar reduction

The presence of reducing sugars in parasite culture media was determined by 3,5-Dinitrosalicylic acid (DNSA) method^54^. Briefly, the cultures were centrifuged for 2000 *×* *g* for 5 min and the supernatant was used for the analysis. To do so, a standard curve with glucose was generated (0.1–5 mg/mL) to quantify the reducing sugars of cell culture supernatants. A volume of 15 mL of each sample was incubated with 1% DNSA in 2 M sodium hydroxide (NaOH) and then boiled for 5 min. Finally, the samples were cooled on ice for 5 min and read in a Multiskan® Spectrum spectrophotometer at 540 nm.

### Transmission Electron Microscopy

The following samples were used for the TEM analysis: i) promastigotes; ii) haptomonad biofilms (either sections of the cell line from flasks using a wooden handle to fix the flask and further cut with a fine-toothed hacksaw or detached biofilms using rubber policeman cell scrapers (Avantor)); iii) dissected bee guts. These samples were processed as previously described^32^ and analyzed in a Carl Zeiss SMT LIBRA 120 PLUS microscope at Centro de Instrumentación Científica (CIC) of the University of Granada.

To confirm the purity and the structure of the EPS, the samples were analyzed using the procedure previously described^32^. Briefly, 15 μL of each sample was adsorbed for 15 min directly onto Formvar/carbon-coated grids, washed twice in PBS and then stained with 2% (v/v) uranyl acetate (Merck) for 1 min. Then, the samples were left overnight at 4 °C with a soft fixation in PBS with glutaraldehyde 2%, paraformaldehyde 1% as fixatives. Finally, grids were dried at room temperature for 30 min and analyzed in a Carl Zeiss SMT LIBRA 120 PLUS microscope at Centro de Instrumentación Científica (CIC) of the University of Granada.

### Scanning Electron Microscopy

The following samples were used for SEM analysis: i) promastigotes adhered to poly-L-lysine-coated coverslips; ii) haptomonad-like biofilms sections from cell culture flasks; iii) purified soluble EPSs and iv) dissected bee guts. The samples were fixed for 24 h at 4 °C in Karnovsky fixative (2.5% glutaraldehyde and 2% paraformaldehyde in 0.1 M cacodylate buffer (pH 6.9)) and then transferred to the washing solution (0.1 M cacodylate buffer with 2.7% glucose) and kept at 4 °C. Next, the samples were dehydrated in a graded series of ethanol, desiccated using a critical point dryer (LEICA EM CPD 300) and then evaporated with high vacuum carbon coater (EMITECH K975X) and observed in a ZEISS Supra 40VP high-resolution SEM microscope at Centro de Instrumentación Científica (CIC) of the University of Granada.

### Cell cycle analysis

A total of 1 × 10^7^ promastigote forms and detached EHB and LHB biofilms were washed in PBS and fixed with ice-cold 70% ethanol. Next, the parasites were washed and labeled with 40 μg/mL propidium iodide (PI, Thermo Fisher Scientific, USA) plus 10 μg/mL RNAse A (Merck) for 45 min at 37 °C in the dark. Flow cytometry was performed using a Becton Dickinson FACS Canto II flow cytometer (BD Bioscience, San Jose, CA) equipped with an air-cooled 488-nm solid state laser, and a 633-nm HeNe laser. PI was excited with the 488 nm laser and collected through 585/42 LP556 nm band pass (BP) filter and a number of 10.000 events.

### Reactive Oxygen Species (ROS) quantification

The analysis of ROS in the different parasite forms was carried out using 2’,7’-dichlorodihydrofluorescein diacetate (H_2_DCFDA) (Thermo Fisher Scientific) fluorescent dye as described before^55,56^. Briefly, Promastigotes, EHB and LHB (1 × 10^7^ parasites/mL) forms were labeled with 10 µM H_2_DCFDA in the dark for 45 min. As positive controls, the parasites were pre-treated with 100 µM hydrogen peroxide (H_2_O_2_) for 1 h before the labeling with H_2_DCFDA. The fluorescence was determined in a Becton Dickinson FACS Canto II flow cytometer (BD Bioscience, San Jose, CA) equipped with an air-cooled 488-nm solid-state laser. The H_2_DCFDA was excited with the 488 nm laser and collected through 530/630 nm BP. Results analyzed in BD Facs Diva 9.1. software (BD biosciences).

### Cell viability of *L. passim* developmental stages

To evaluate the ratio of live/dead cells of the different *L. passim* developmental stages, the cells were labeled with 5 μM of Syto 9 (Thermo Fisher Scientific) plus 40 μg/mL PI and incubated for 30 min at room temperature. Flow cytometry was performed using a Becton Dickinson FACS Symphony flow cytometer equipped with an air-cooled 488-nm solid state laser, and a 633-nm HeNe laser. Syto 9 was excited at 488 nm solid-state laser and collected through 530/630 nm BP. PI was excited with the 488 nm laser and collected through 585/42 LP556 nm BP filter and a number of 10.000 events.

### Stress resistance assays

To evaluate the increased resistance of monoxenous trypanosomatids compared with human-infective trypanosomatid parasites to harsh conditions, two stresses (thermal and osmotic) were selected. We compared the performance of *L. passim* Promastigotes, EHB, LHB forms and *L. pyrrochoris* promastigote, *L. major* promastigote and *T. cruzi* epimastigote forms as EPS non-secreting control parasites. The cells were exposed to hypoosmotic shock (distilled water) and freezing stress (4 °C and −20 °C) for 0 h (T0), 24 h (T1), 72 h (T2) and 144 h (T3). For hypoosmotic shock, the different parasite forms were washed three times with PBS and incubated in sterile distilled water and for freezing stress the cultures were storaged at 4 °C and −20 °C (without cryoprotective agents) and then incubated for 30 min at 28 °C prior to the addition of resazurin. The viability of the cells was then calculated by incubating the parasites with 16 µM resazurin (Merck & Co., USA) for 4 h followed by the measurement of the reduction of the dye at 579 nm and 600 nm in a Multiskan Spectrum (Thermo Fisher Scientific) spectrophotometer. Besides, an aliquot of 100 μl of the cultures was seeded 24-well plates (Nunc) in 2 ml of fresh BHT 5% iFBS media and the recovery evaluated by light microscopy after the different treatments.

### Proteomic analysis of *L. passim* EPS

The proteome composition of the EPS fractions was analyzed using *L. passim* latelog phase promastigote forms (1–2 × 10^7^ cells/ml). At this concentration, the cell cultures were centrifuged at 2000 *×* *g* for 10 min and pellets washed twice in PBS. Then, the parasites were resuspended in BHT media without serum at a concentration of 1 × 10^8^ cells/ml and incubated for 16 h to induce the secretion of EVs as previously described^25^. Next, the cultures were centrifuged at 2000 *×* *g* and the supernatants filtered through a 0.45 μm pore filter (Sartorius). Finally, the filtered media was ultracentrifuged at 100,000 × *g* for 4 h to obtain EPS. All the steps were performed in an ultracentrifuge Avanti J-301 (Beckman Coulter, USA) with a JA-30.50 Ti rotor. The resulting pellet was washed three times in PBS in an ultracentrifuge Sorvall WX80 (Thermo Fisher Scientific) with F50L-24 ×1.5 fixed-angle rotor and resuspended in 100 μL PBS. Ultrastructural details of the sample were characterized by TEM, and particle size distribution analyzed by Nanoparticle Tracking Analysis (NTA).

The protein concentration, digestion and Matrix-assisted laser desorption/ionization-time of flight mass spectrometry (MALDI-TOF MS) analysis was realized at the Centro de Biología Molecular Severo Ochoa (CBMSO) as previously described^25^. Next, a database search was performed (Uniprot-*Leishmania major* Friendlin strain) and protein enrichments were classified according to KEEG metabolic pathway and Gene Ontology (GO) enrichment tools in the TritrypDB database (https://tritrypdb.org/tritrypdb/app). A cut-off value of *p* ≥ 0.01 was used. The proteome analysis were performed in triplicate.

### Experimental infections of honeybees

Infection of 2-days worker bees with latelog phase promastigote forms (0.5–1 × 10^7^ cells/ml) were performed as previously described^39^. Briefly, newborn honey bees were collected from an uncapped frame and were kept in cages with *ad libitum* food in an incubator (33 °C) until infection. At an age of 2 days, honey bees were manually fed with 2 μl of PBS containing either 1 × 10^4^ promastigotes/bee (i.e low dose) or 6 × 10^5^ promastigotes/bee (i.e. high dose). Control bees received 2 μl of PBS. As microscopic sample preparation can entail loss of trypanosomatids, honey bees were re-infected with the same dose two and five days after the first artificial infection as previously described^11^. Four cages of 15 bees each were prepared for each treatment. Bees were kept at 28 °C in an incubator with *ad libitum* access to 50% sucrose syrup +2% Promotor L (Calier Lab, Spain). At day six after the first artificial infection, 3-4 alive bees were collected from each cage to seek *L. passim* in either their feces or their gut, respectively, in SEM analysis. Feces were recovered by gently pressing the bee abdomen with pipette tips on a Petri Dish. The material was resuspended using 100 μl of PBS. Fecal samples were checked on a light microscope for the presence of *L. passim*, and promastigote forms were counted in a Newbauer chamber and total number of parasites per fecal sample calculated. The gut of each honey bee was pulled from the sting, washed in PBS and stretched in a 0.45 μm cellulose nitrate filter (Sartorius Biolab, Germany). Samples were fixed for 24 h at 4 °C in Karmovsky fixative (2.5% glutaraldehyde and 2% paraformaldehyde in 0.1 M cacodylate buffer (pH 6.9)), washed in 0.1 M cacodylate buffer with 2.7% glucose and kept at 4 °C. Then, the ileum and rectum from gut samples were dissected and a longitudinal scission was made with a scalpel to let the intestinal tract open and processed for SEM analysis as previously described^31^.

### Statistical analysis

Results are given as mean values ± standard deviation (SD). Significant differences among the different parasite forms were analyzed using Two-way analysis of variance (ANOVA) and Tukey´s post hoc test. *P* < 0.05, results were considered statistically significant. The statistical analyses were performed using GraphPad Prism 9.0.2 software.

### Reporting summary

Further information on research design is available in the Nature Research Reporting Summary linked to this article.

### Supplementary information

Supplementary information Promastigote EPS secretion and haptomonad biofilm formation as evolutionary adaptations of trypanosomatid parasites for colonizing honeybee host

Reporting Summary

## Data Availability

The data supporting the findings of this study are available within the paper, its Supplementary Information, or Source Data available via ProteomeXchange with identifier PXD040355 (10.6019/PXD040355). Spectrophotometric raw data was deposited in Zenodo: 10.5281/zenodo.10245615.
