# Supplementary material for: Promastigote EPS secretion and haptomonad biofilm formation as evolutionary adaptations of trypanosomatid parasites for colonizing honeybee hosts
Source: NPJ Biofilms Microbiomes. 2024 Mar 21;10:27. doi: 10.1038/s41522-024-00492-x (PMC10957890; doi:10.1038/s41522-024-00492-x)
Supplement: Supplementary file 1 — Supplementary information Promastigote EPS secretion and haptomonad biofilm formation as evolutionary adaptations of trypanosomatid parasites for colonizing honeybee host [file 41522_2024_492_MOESM1_ESM.pdf]

Supplementary information for

Promastigote EPS secretion and haptomonad biofilm formation as evolutionary adaptations of trypanosomatid parasites for colonizing honeybee hosts.

Jéssica Carreira de Paula, Pedro García Olmedo, Tamara Gómez-Moracho, María Buendía Abad, Mariano Higes, Raquel Martín Hernández, Antonio Osuna, Luis Miguel de Pablos Torró.

Corresponding author: Luis Miguel de Pablos Torró (lpablos@ugr.es)

Supplementary Fig. 1. Nanoparticle Tracking Analysis of EPS-EVs purified using ultracentrifugation.

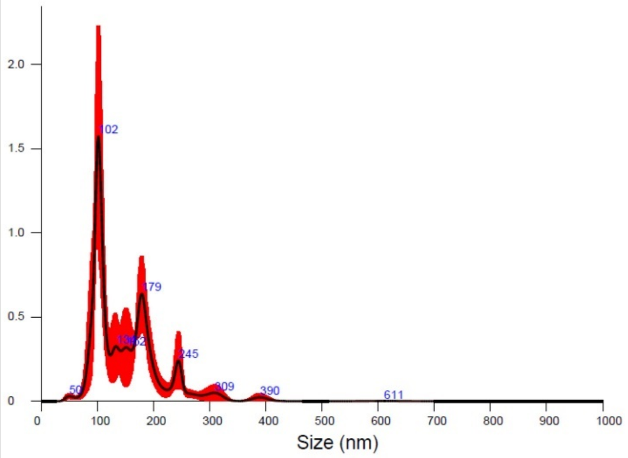

Averaged FTLA Concentration / Size for Experiment:
EPS Filtrado. Dilucion 4 2023-12-01 13-29-53
Error bars indicate + / -1 standard error of the mean

Results

Stats: Merged Data	
Mean:	149.6 nm
Mode:	101.7 nm
SD:	62.1 nm
D10:	93.7 nm
D50:	133.1 nm
D90:	236.7 nm
Stats: Mean +/- Standard Error	
Mean:	150.7 +/- 5.5 nm
Mode:	99.7 +/- 3.4 nm
SD:	62.2 +/- 8.6 nm
D10:	93.4 +/- 3.4 nm
D50:	142.0 +/- 11.5 nm
D90:	230.0 +/- 13.6 nm
Concentration (Upgrade):	7.31e+07 +/- 8.62e+06 particles/ml
	7.0 +/- 0.4 particles/frame
	9.0 +/- 0.8 centres/frame

Concentration measurements may require some caution due to noise
See summary file for more info

The graph at the left indicates size distribution of the nanoparticles found in the EPS. The samples were obtained by differential centrifugation with a last step of ultracentrifugation at 100.000 x *g*. Note the heterogenous size of the EPS found in the sample with a maximum peak at 102 nm. The mean size was of 149.6 nm and the mode at 101. 7 nm. The D10/D50/D90 values were 93.4 ± 3.4/142 ± 11.5/230 ± 13.6 nm. D10 is the point in the size distribution where 10% of the sample is contained, D50 is the point where 50% of the sample is contained (median), and D90 is the point where 90% of the sample is contained. SD: Standard deviation; CV: Coefficient of variation.

Supplementary Fig. 2. Structural details of the *L. passim* promastigote EPS.

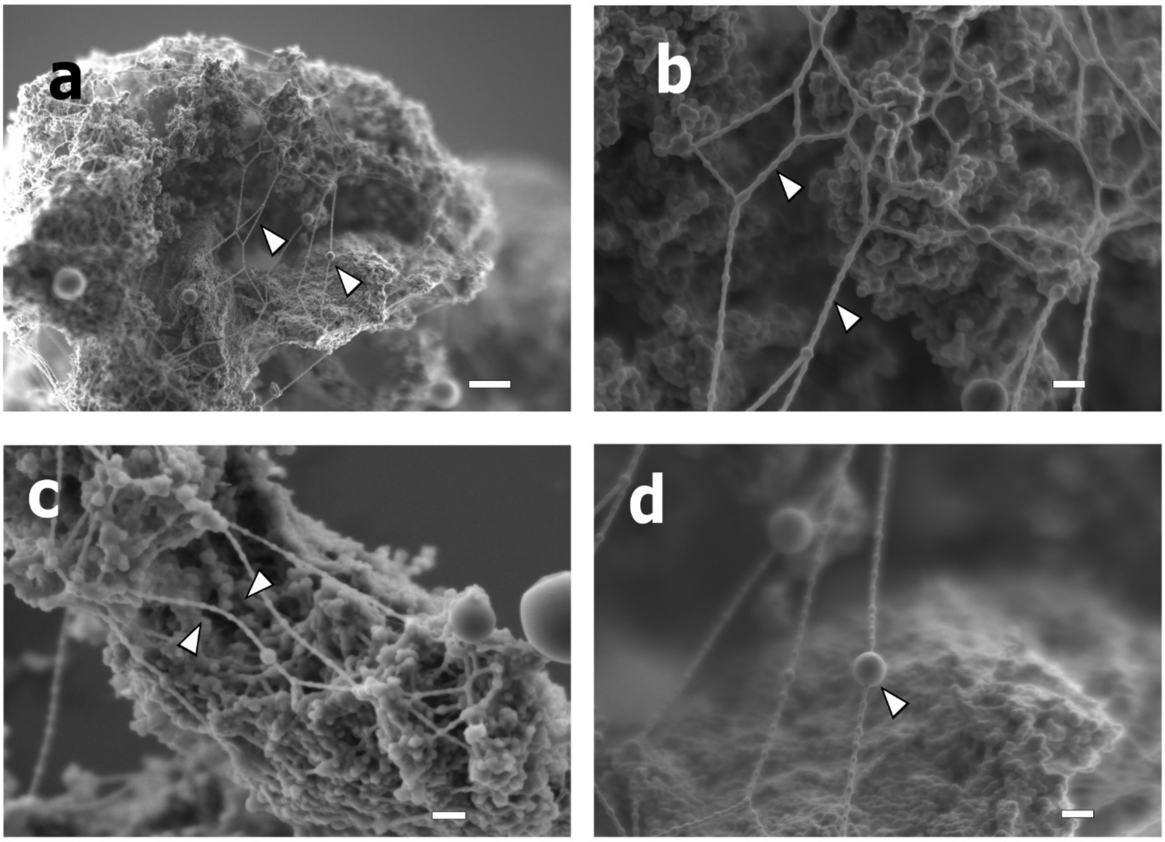

EPSs analysed by Scanning Electron Microscopy. **a, b, c** Different magnification of EPS showing a thick mass of polymeric material surrounded by long fiber projections of spherulitic material forming “rosary beads” (white head arrows). Scale bars represents 1 µm, 200 nm and 200 nm respectively. **d** Fibers are eventually bound with spherulites of higher dimensions (white head arrow). Scale bar represents 200 nm.

Supplementary Fig. 3. Proteome of *L. passim* EPS.

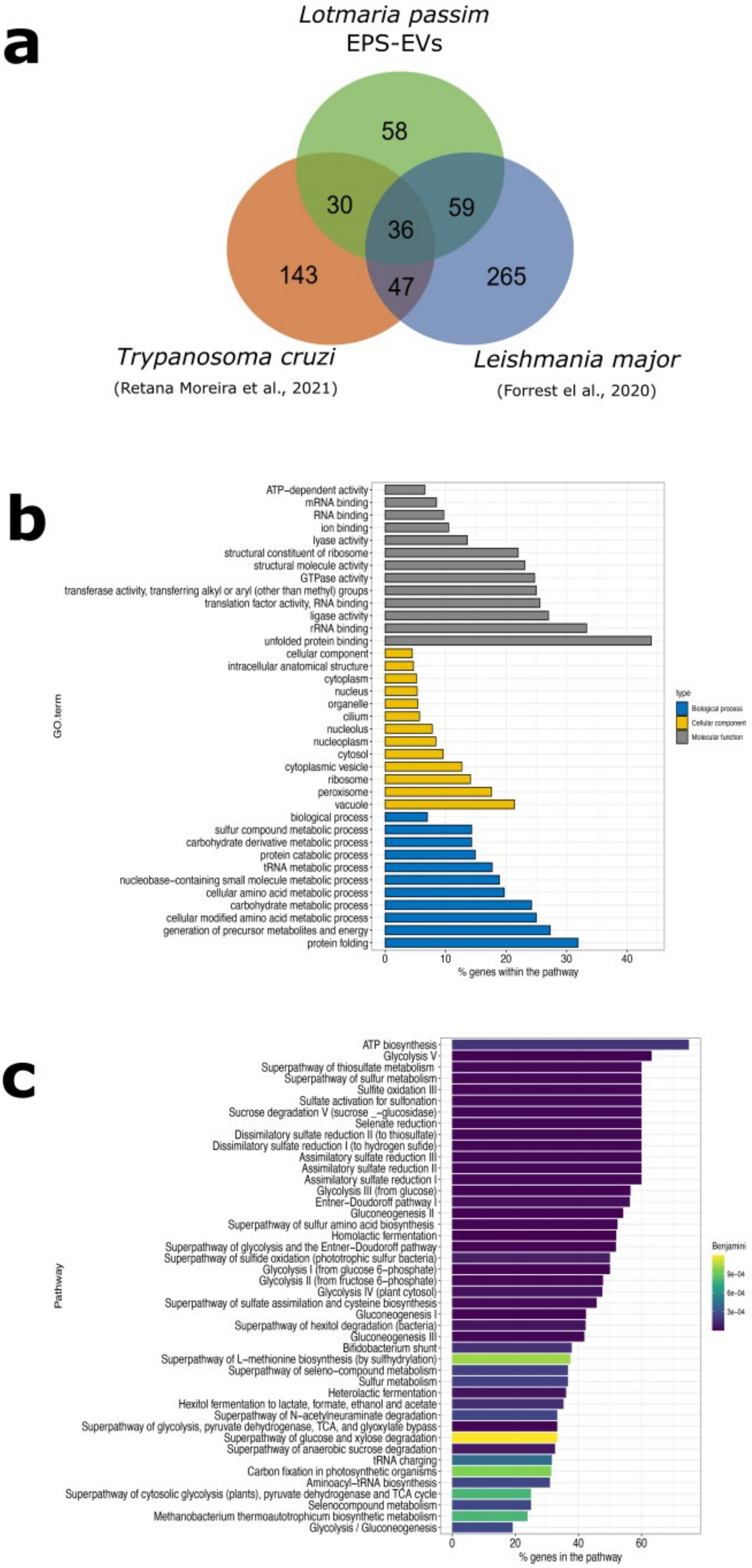

a Venn diagram of the protein content of *L. passim* EPS-EVs vs *Leishmania major*[32] and *Trypanosoma cruzi*[33] EVs. **b** GO term enrichment ($P < 0.05$) of *L. passim* EPS-EVs for Biological process, Molecular function and Cellular component. **c** KEEG Metabolic Enrichment Pathways of the full EPSs dataset. The significance ($P < 0.05$) was color coded using Benjamini-Hochberg False discovery rates.

Supplementary Fig. 4. Structural details of the *L. passim* haptomonad-like biofilms.

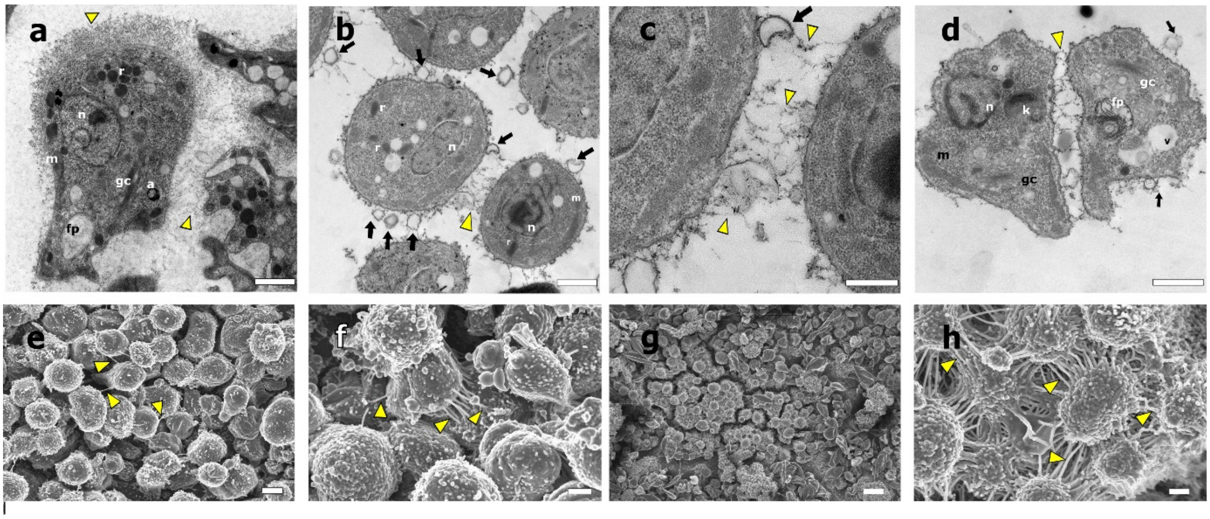

a, b, c, d Transmission Electron Microscopy analysis of haptomonad cells. Note the cloud of tightly bound EPSs at the surface of detached (**a**), and attached (**b, c, d**), haptomonad cells. Scale bars represents 1 µm, 1 µm, 300 nm and 1 µm respectively. **e, f, g, h** Haptomonad-like biofilms analysed by Scanning Electron Microscopy. Yellow head arrows indicated long fiber projections of EPSs binding individual cells, and black arrow indicated spherulites**.** Scale bars represents 1µm, 1µm; 6 µm and 600 nm respectively.

Supplementary Fig. 5. *C. mellificae* trypanosomatid parasites are also capable of forming biofilms *in vitro*.

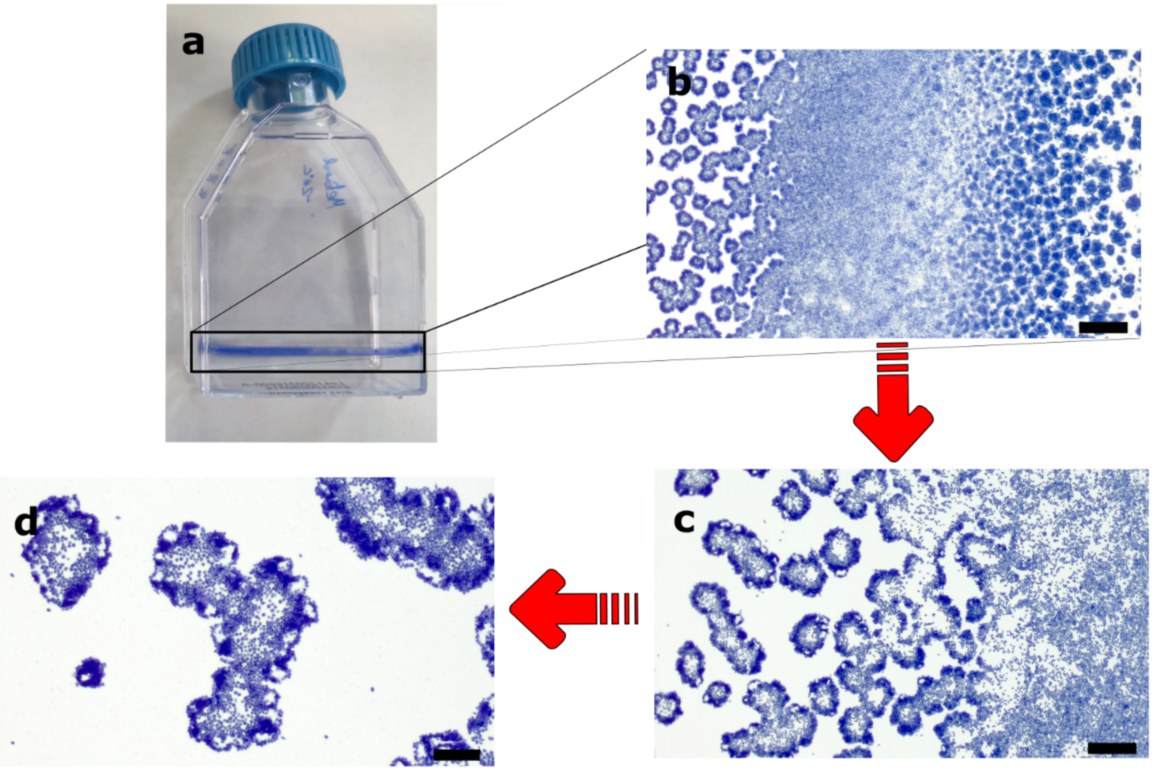

a *C. mellificae* cell culture flask stained with Giemsa stain at the stationary phase of the culture. Note the layer at the air/liquid interphase in cell culture flasks. **b, c, d** images show different magnifications of the biofilm colonies formed by *C. mellificae*. Scale bars represents 100 µm, 100 µm and 50 µm respectively.

Supplementary Fig. 6. Scanning electron microscopy analysis of *L. passim* life cycle stages in experimental infections of honeybees.

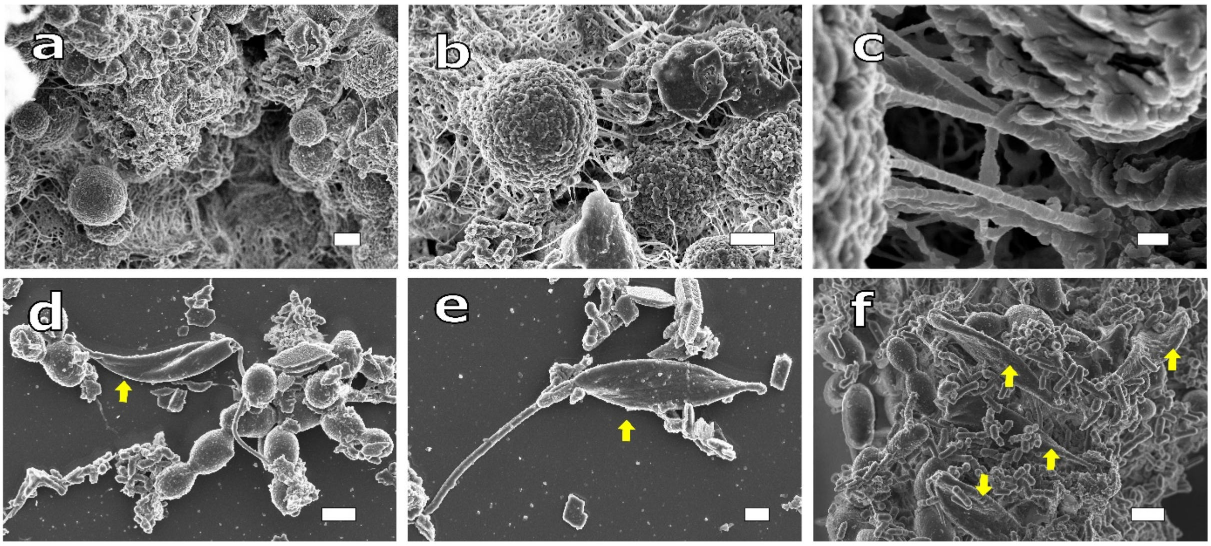

a, b Haptomonad biofilms are formed over the surface of the honeybee hindgut. **c** EPS fibers attaching two contiguous haptomonad cells. Scale bars represents 2 µm, 1 µm and 200 nm respectively. **d, e** the fecal content of infected honeybees showing *L. passim* promastigote forms (yellow arrow) with elongated flagella surrounded by bacteria and yeast. Scale bars represents 2 µm and 1 µm respectively. **f** A dense fecal mass with promastigote forms closely ligated and cemented with released yeast and bacteria. Scale bar represents 2 µm.

Supplementary Fig. 7. Infected ileum with haptomonad biofilms of *L. passim*.

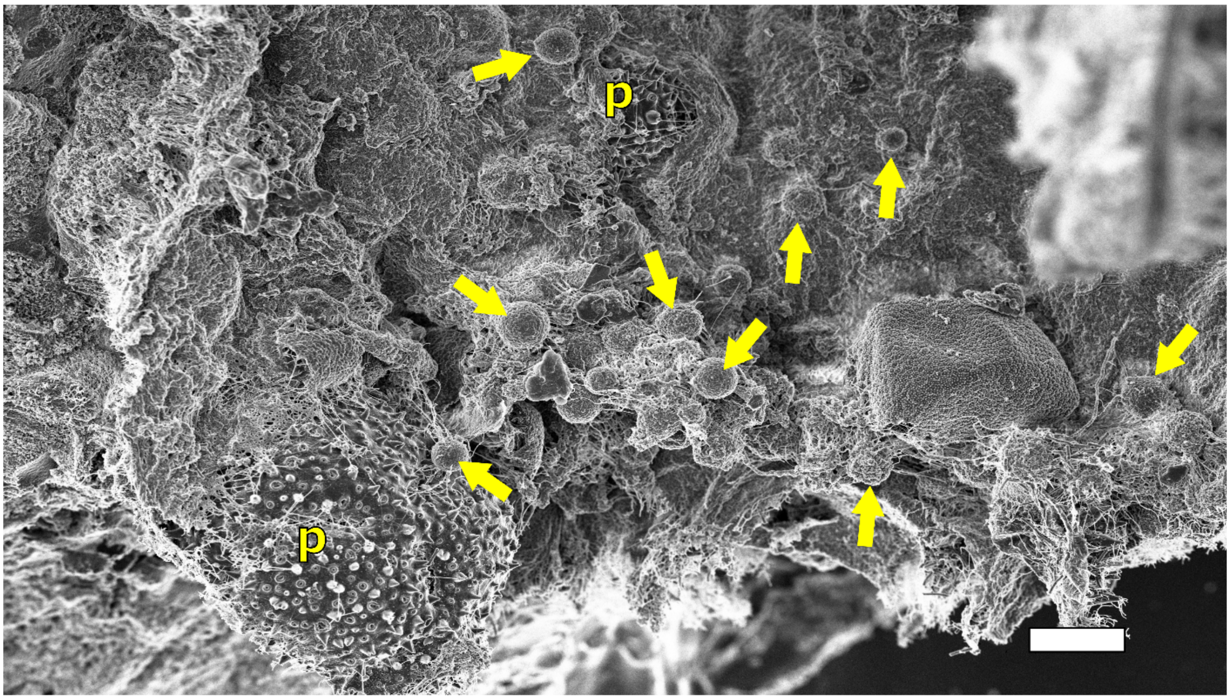

The image shows the widespread location of haptomonad forms colonizing the surface of honeybee ileum. The parasites were adhered both individually and as biofilm microcolonies covered by dense EPS matrixes. Yellow arrows indicate the presence of haptomonad cells over. P: pollen. The scale bar represents10 µm.

Supplementary Fig. 8. Examples of cell cycle (left) and ROS resistance measurements (right) histograms in the different life cycle stages (promastigotes, early haptomonad-like biofilms and late haptomonad-like biofilms) of *L. passim* parasites.

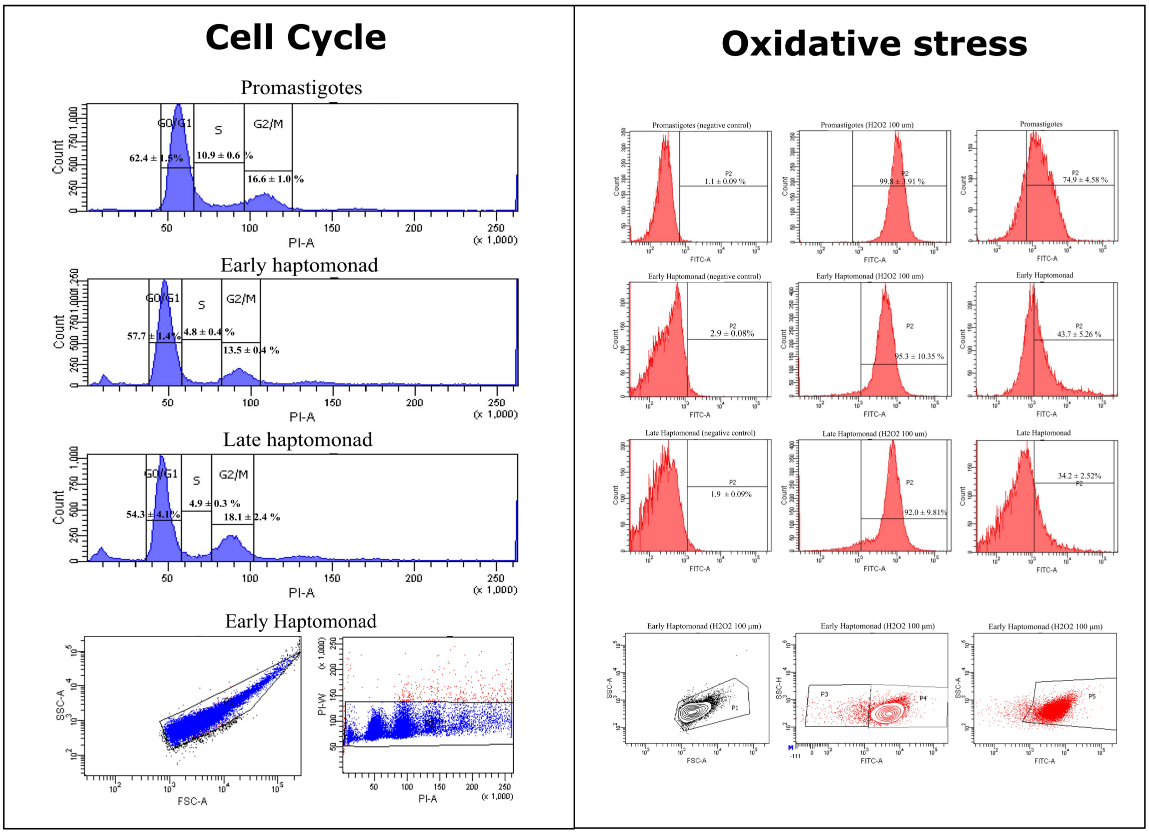

For cell cycle (left panel), propidium iodide staining was used to check the DNA quantity on cells in the different phases of the cell cycle. In the image above an example of the gating performed to generate SSC/FSC and PI plots. For flow cytometric measurement of reactive oxygen species (right panel) H_2O_2 was used ROS inducer and H_2DCFDA as an indicator for ROS. The first column at the left represents histograms are non H_2O_2 treated cells (negative controls), the central column represents cells treated with H_2O_2 (positive control) and the right column the oxidative stress percentage in each of the forms analysed. In the image above an example of the gates performed to generate SSC/FSC and FITC/SSC plots. For both cases same number of cell events were gated and results analysed in BD Facs Diva 9.1. software (BD biosciences).

Supplementary Fig. 9. Examples of Live/dead flow cytometric analysis in the different life cycle stages (Promastigotes, Early haptomonad-like biofilms and Late haptomonad-like biofilms) of *L. passim* parasites.

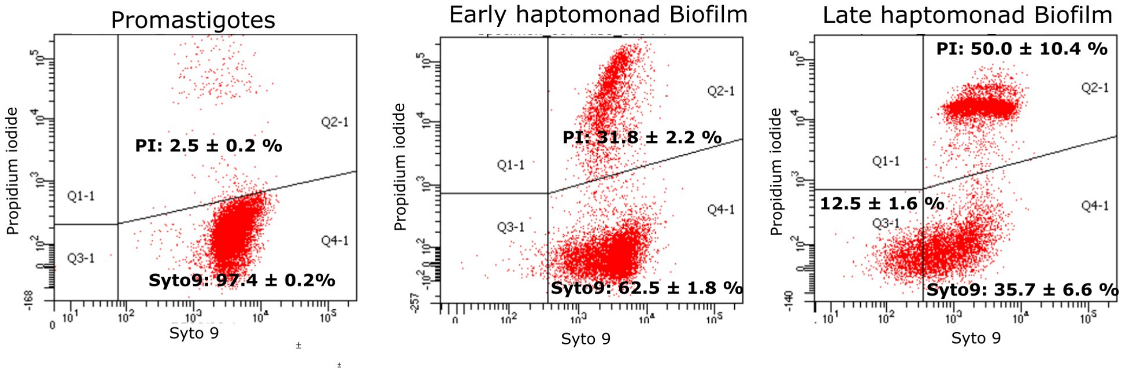

For this analysis, cells where co-stained with propidium iodide indicating dead cells (upper right square) and Syto 9 indicating live cells (bottom right square). Note that the gated population in the upper right square (propidium iodide (dead cells)) increases towards late haptomonad-like biofilms.

Supplementary Fig. 10. Giemsa stain of promastigote cell cultures of *L. pyrrochoris* C1 strain.

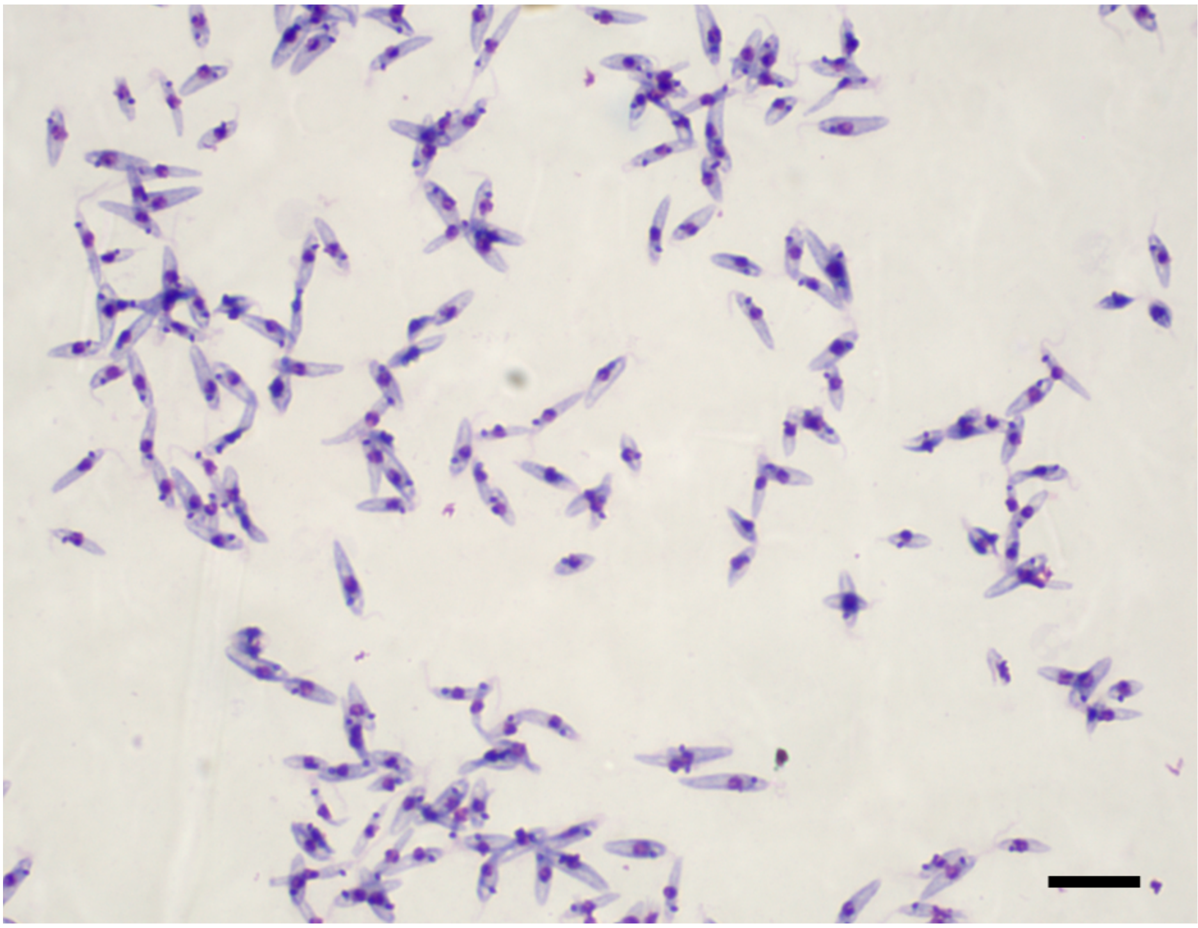

L. pyrrochoris cells have a tipical trypanosomatid liberform conformation with a free flagellum emerging from the flagellar pocket. Scale bar represents 10 µm.

Supplementary Fig. 11. Stress resistance of promastigotes, early haptomonad-like biofilms, and late haptomonad-like biofilms in *L passim* and *C. mellificae*.

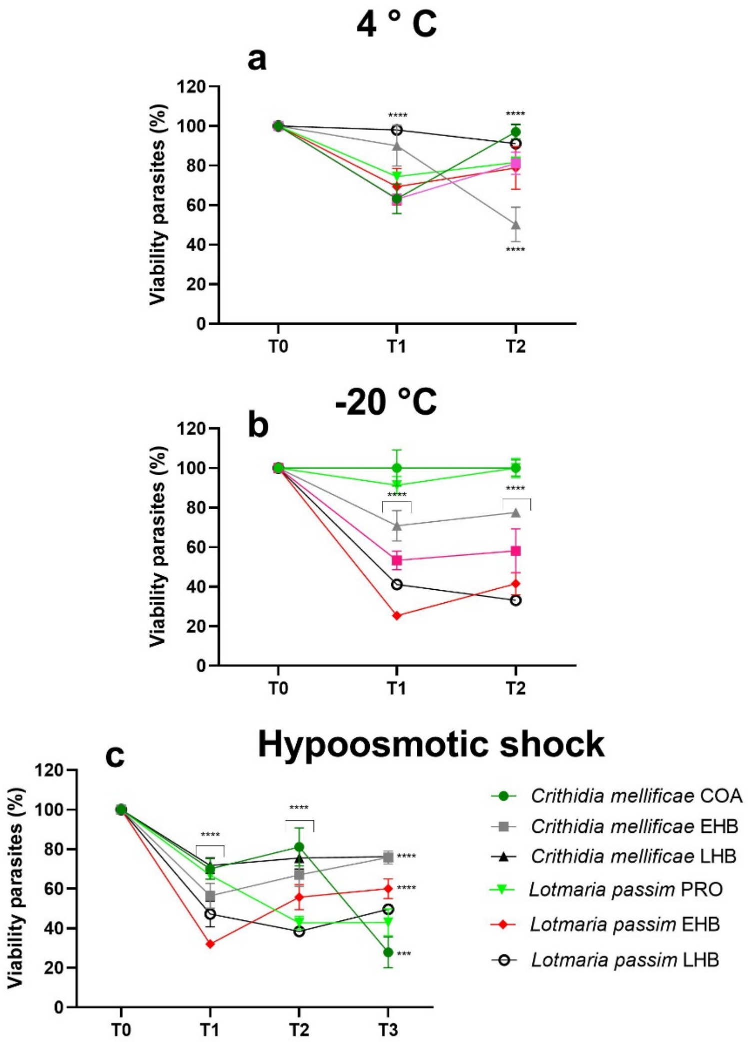

a, and **b,** Viability of the different forms of *L. passim* and *C. mellificae* at 24 h (T1) and 72 h (T2) after cold-shock treatments at 4, and -20 degrees. The percentage of viability was obtained by comparing with non-treated cells (T0). **c** Viability of the different forms at 24 h (T1) and 72 h (T2) and 144 h (T3) after hypoosmotic shock. The viability of the parasites after treatments was measured spectrophotometrically using resazurin and the percentage of metabolic reduction and transformation into resofurin compared with non-treated cells (T0). Two-way ANOVA followed by Tukey´s post hoc test was performed. **** $P \leq 0.0001$; ***$P \leq 0.0004$. Data is presented as mean ± s.d.
